# In silico evolution of globular protein folds from random sequences

**DOI:** 10.1073/pnas.2509015122

**Published:** 2025-06-30

**Authors:** Harutyun Sahakyan, Sanasar G. Babajanyan, Yuri I. Wolf, Eugene V. Koonin

**Affiliations:** ^a^Computational Biology Branch, Division of Intramural Research, National Library of Medicine, NIH, Bethesda, MD 20894

**Keywords:** protein folds, protein evolution, protein structure prediction, fold nucleation, molecular dynamics

## Abstract

The origin and evolution of protein folds are among the most challenging, long-standing problems in biology. We developed Protein Fold Evolution Simulator (PFES), a computational approach that simulates evolution of globular folds from random amino acid sequences with atomistic details. PFES introduces random mutations in a population of protein sequences, evaluates the effect of mutations on protein structure, and selects a new set of proteins for further evolution. Iteration of this process allows tracking the evolutionary trajectory of a changing protein fold that evolves under selective pressure for protein fold stability, interaction with other proteins, or other features shaping the fitness landscape. We employed PFES to show how stable, globular protein folds could evolve from random amino acid sequences as monomers or in complexes with other proteins. The simulations reproduce the evolution of many simple folds of natural proteins as well as emergence of distinct folds not known to exist in nature. We show that evolution of small globular protein folds from random sequences, on average, takes 1.15 to 3 amino acid replacements per site, depending on the population size, with some simulations yielding stable folds after as few as 0.2 replacements per site. These values are lower than the characteristic numbers of replacements in conserved proteins during the time since the Last Universal Common Ancestor, suggesting that simple protein folds can evolve from random sequences relatively easily and quickly. PFES tracks the complete evolutionary history from simulations and can be used to test hypotheses on protein fold evolution.

## Significance

Origin of protein folds is an essential early step in the evolution of life that is not well understood. We address this problem by developing a computational framework approach for protein fold evolution simulation (PFES) that traces protein fold evolution in silico at the level of atomistic details. Using PFES, we show that stable, globular protein folds could evolve from random amino acid sequences with relative ease, resulting from selection acting on a realistic number of amino acid replacements. About half of the in silico evolved proteins resemble simple folds found in nature, whereas the rest are unique. These findings shed light on the enigma of the rapid evolution of diverse protein folds at the earliest stages of life evolution.

The origin of life, one of the most important problems in all of science, is closely connected with the origin and evolution of proteins that are central to all biological processes (1). Proteins have the most diverse functions among biological macromolecules and are involved in all cellular processes. The function of any protein critically depends on its structure, or fold, which is formed by folding the linear amino acid chains into a specific three-dimensional shape (2–4). Origin and evolution of protein folds remain extremely challenging problems because the methodology required for thorough exploration of evolutionary trajectories is still lacking despite major advances in evolutionary genomics, phylogenetics, bioinformatics, and theoretical biophysics during the last decades. Although many potentially plausible scenarios of protein evolution have been proposed, these are primarily based on simplified, in particular, lattice models that adopt reduced amino acid alphabets and cannot be directly tested experimentally (5–7). Nevertheless, many hypotheses seem to converge on the scenario of proteins originating as small peptides with random sequences—or possibly, quasi-random sequences that were preadapted for activities such as RNA binding or metal chelation that did not require folding—and gradually evolving into more complex structures with distinct folds (8–16). A detailed scenario has been proposed in which proteins would evolve in the primordial RNA world when ribozymes using amino acids and short peptides as cofactors gained peptidyl transferase activity, and gradually, this mechanism evolved into the translation system (17). An alternative hypothesis, based on a simplified computational model of polar and nonpolar monomers, posits that at the earliest stages of protein evolution, small random peptides formed foldamers, which had the capacity to interact and elongate by self-catalyzing peptide bond formation (18).

Until recently, simulation of protein evolution with all-atom models aiming at testing such hypotheses was not feasible because of the lack of fast and accurate approaches for protein structure prediction. The development of machine learning-based tools for fast and robust protein structure prediction including AlphaFold, RoseTTAFold, and ESMfold has changed this situation (19–22). Taking advantage of these protein structure modeling methods, we developed protein fold evolution simulator (PFES), a toolkit to simulate and analyze protein fold evolution with atomistic details under different conditions. Using PFES, we show how a protein fold could evolve from random sequences as a monomer or in a complex by interacting with another protein. From multiple protein evolution simulations, we provide an estimate of how many mutations are required for a protein fold to nucleate from a random sequence. Our results suggest that, at the transitional stage of evolution between an RNA-peptide world and modern-type biological systems encompassing diverse proteins, stable protein folds could evolve from random sequences relatively easily and quickly.

## Results

### Protein Fold Evolution Simulation.

**Fig. 1.**
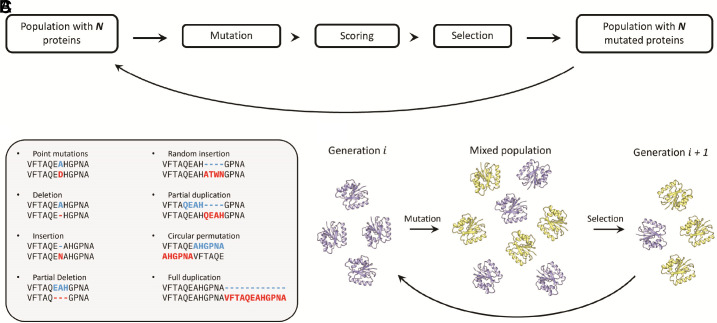
Workflow of protein fold evolution simulation. (*A*) Each protein in a population mutates, and the effect of each mutation on protein structure is evaluated to calculate a fitness score; the next generation of proteins is selected based on the fitness scores. (*B*) Possible mutation types that occur during the simulation. (*C*) Mutation and selection mechanism. All proteins in the population mutate, creating a mixed population of size **2*****N*** from which the next generation is selected.

The main design of PFES is straightforward: i) random (or quasi-random) mutations are introduced into a population of polypeptide sequences, ii) the effect of mutations on protein structure is evaluated, and fitness scores are calculated, and iii) a subset of polypeptides from the given generation is selected for the next iteration (Fig. 1*A*). By iteratively introducing and evaluating thousands of mutations in a population of evolving random amino acid sequences, one can observe how protein structure nucleates and changes through large-scale conformational rearrangements, switching from onefold to another. The simulation starts with a population of size ***N*** that can be created by generating ***N*** random sequences or mutating one random or predefined sequence ***N*** times to create ***N*** mutated variants of the initial sequence. Possible mutations and probabilities for each mutation are provided in several “evolutionary dictionaries” that contain information about allowed mutations and their probabilities (see *Methods* for details).

The probability of amino acid substitutions can be uniform with flat rates, that is, any substitution is equally probable. Alternatively, the substitution probabilities can be defined by the codon frequencies or the occurrence of amino acids in natural proteins or in any other manner, for example, using substitution matrices such as PAM (Point Accepted Mutation) or BLOSUM (BLOcks SUbstitution Matrix) (23, 24). In addition to the amino acid substitutions, several types of nonpoint mutations affecting protein length are allowed, including single amino acid insertion or deletion, multiple amino acid deletion, insertion of several random amino acids, partial or complete duplications, and circular permutations (Fig. 1*B*).

The effect of each mutation is evaluated at the next step, based on the structure prediction with ESMfold (21). Although any other method can be used in this workflow, ESMfold has a good tradeoff between speed and accuracy, allowing simulation of multiple generations of proteins where changes in the protein fold or other potentially important events can be observed. The final fitness score, calculated for each protein in the population, is a multiplicative function consisting of several terms (*Methods*) that reflect the predicted model quality and fold stability, which is one of the key constraints in protein evolution because most proteins require a stable fold to be functional, and moreover, many unfolded proteins are toxic to the cell (even though intrinsically disordered proteins can perform a variety of biological functions and might have been important at the early stages of the evolution of life) (25–27).

By introducing mutations into all sequences in the population, we create a mixed population of size **2*N*** containing the original and mutated sequences (Fig. 1*C*). For the next generation, ***N*** proteins are selected via weak or strong selection. In the strong selection mode, ***N*** proteins with the highest fitness score are passed from generation ***i*** to ***i* + 1**, that is, only the fittest half of the mixed population survives to form the population of the next generation. Although strong selection is relatively rarely observed in biological evolution, it has helpful application in the PFES framework for quickly optimizing the already evolved proteins by maximizing their fitness scores. The weak selection mode represents a more plausible evolutionary process with stochastic selection, which allows random drift and makes possible deeper exploration of the fold space. The probability of a protein survival, in this case, is proportional to the fitness score and can be additionally regulated by a β-factor to change the selective pressure (*Methods*).

### Evolution of Protein Folds from Random Sequences.

**Fig. 2.**
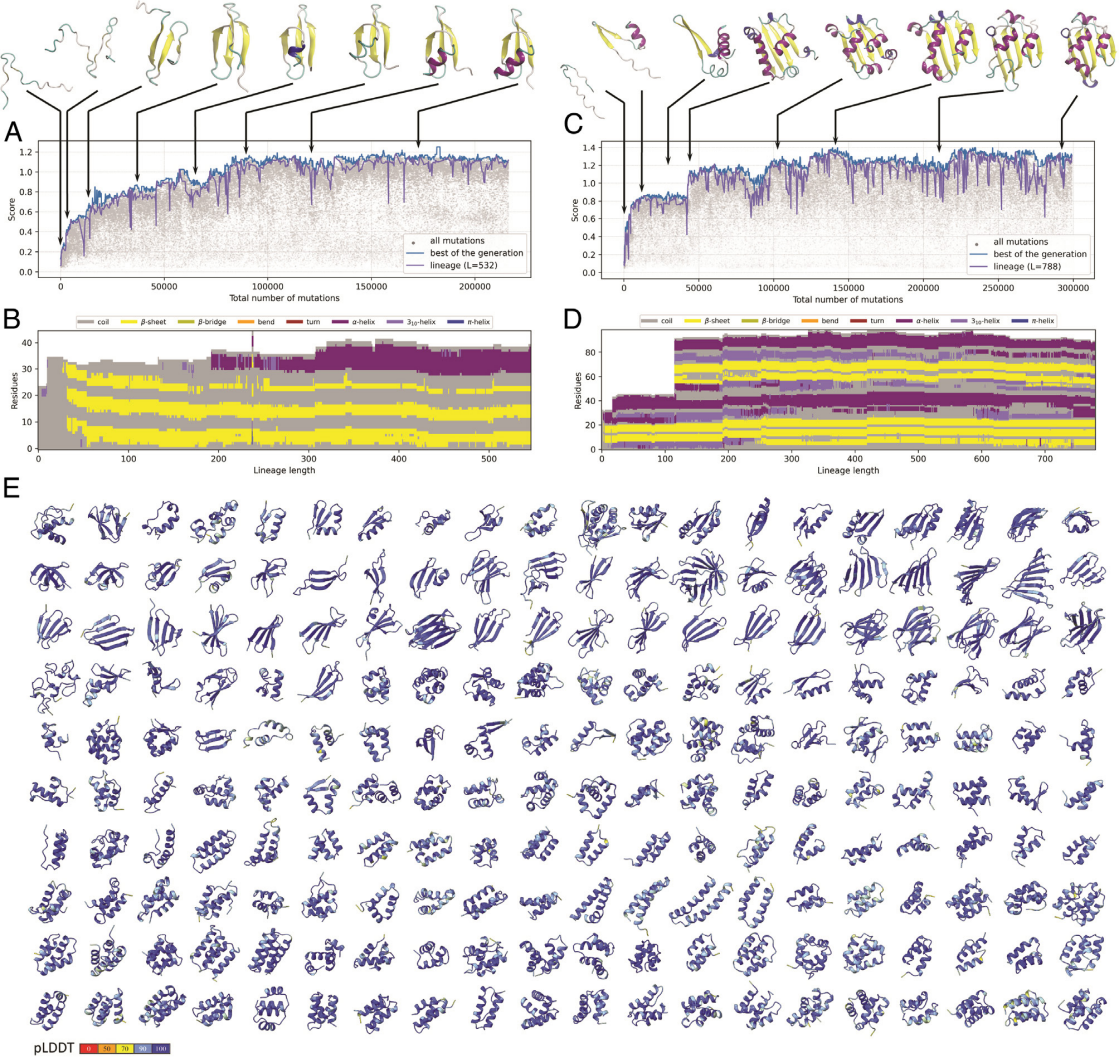
Evolution of protein folds in PFES. (*A*) The fitness score plotted for all sequences (gray), the best sequences in each generation (blue), the lineage of the best sequences from the last generation (violet), and intermediate structures of a gradually evolving protein representing different points of the evolutionary trajectory. (*B*) Changes in the secondary structures after each mutation in the lineage. (*C*) Fitness score and representative structures of a protein evolving via complete domain duplication. (*D*) Corresponding changes in the secondary structures. (*E*) Examples of other structures evolved in simulations. Proteins are sorted by similarity according to the hierarchical clustering (*SI Appendix*, Fig. S2).

A series of simulations with varying parameters were run, representing different scenarios in an attempt to understand how protein folds could evolve. In the simplest case, a simulation in the stochastic (weak) selection mode started from 100 random peptide sequences consisting of 24 amino acids each. The initial set of peptides emerging from 100 different mutations of a random sequence forms the first generation. In the simulation, sequences randomly mutate, and under selection, favorable mutations accumulate, leading to the formation of compact protein structures. The initial random sequences are primarily disordered, with no compact fold predicted, but the fixation of favorable mutations gradually results in the formation of contacts within the evolving peptide, and later, when enough contacts are established, the peptide adopts a distinct fold with defined secondary structure elements. The radius of gyration of evolving proteins is high at the start of the simulations, indicative of extended, disordered structures, which become more compact and globular in the course of evolution (*SI Appendix*, Fig. S1). Typically, the resulting fold is very simple, such as alpha/beta-hairpin, helix–turn–helix (HTH), or WW domain, as illustrated in Fig. 2*A*. As a rule, the first nucleated structure had a low score, indicative of instability of the fold, but as the population evolved, more stable folds originated with higher pLDDT (predicted Local Distance Difference Test) and pTM (predicted template modelling) scores. Once a minimal stable structure is fixed, it goes through small changes where, for hundreds and thousands of mutations, the fold architecture does not undergo major modifications although the sequence can drift and change substantially. This process can be described as the evolving protein reaching a local peak on the fitness landscape (28) where only small and gradual changes in the loops or minor modifications of the secondary structure take place, usually leading to more compact and stable structures. More noticeable changes in the structure are typically associated with small insertions, which also occur mainly in the loops. For example, the C-terminal loop in one of the evolving proteins started forming a small 3_10_ helix that grows and transforms into a short alpha-helix (Fig. 2 *A* and *B* and Movie S1).

Every simulation yields a unique evolutionary trajectory, in each case, resulting in different proteins. The simulations described above were repeated 200 times, and mostly, the evolved protein had a distinct fold, although some generic similarities were observed (Fig. 2*E* and *SI Appendix*, Fig. S2). Clustering of the final folds based on a minimal TM-score of 0.5 showed that the majority of folds (110 of 200) were unique, whereas the remaining ones formed clusters, of which the largest ones consisted of proteins with simple folds, such as HTH, beta-sandwiches, beta-sheets, and alpha or beta-hairpins (*SI Appendix*, Fig. S2). Clustering these proteins at 30% sequence identity yielded only singletons, indicating that the evolving proteins shared virtually no sequence similarity. PSI-BLAST (Position-Specific Iterative Basic Local Alignment Search Tool) search in the NCBI NR database yielded no significant sequence matches among natural proteins except for the protein from simulation #29, which showed significant similarity to some uncharacterized Zn-finger proteins. Protein folds consisting mostly of alpha helixes were most common in our simulations (*SI Appendix*, Fig. S3*A*), which can be explained by the mechanism of their evolution. Once a small alpha helix is nucleated, it continues to grow gradually with insertions at the termini or in the middle of the helix until a critical length is reached. Duplications often lead to the formation of more complex structures, such as an alpha-hairpin from a single helix or a helical bundle from an alpha-hairpin. In evolutionary simulations, simple beta folds also reoccurred multiple times, and evolution of these folds often followed a distinct pattern. A beta-hairpin evolved from random sequences duplicated fully or partially several times, forming a larger beta-sheet, which then folded into a beta-sandwich or jelly-roll-like structure (*SI Appendix*, Fig. S4). In some cases, this process leads to the evolution of beta-barrel-like structures. For example, the proteins from simulations #188 and #197 produced the SH3 fold, an ancient domain that is widespread in all life forms, particularly, among translation system components (29).

Structural search with Foldseek (30) in PDB, AlphaFold/UniProt50, and MGnify-ESM30 databases yielded multiple hits with 0.8 or greater coverage of the queries and mainly partial coverage of targets. This structural search showed that 82 of the 200 structures evolved in the simulations had structural analogs among natural proteins, with a Foldseek probability of at least 0.95, including 23 hits from PDB (*SI Appendix*, Table S1) (31). The largest number of hits of the structural search involved simple beta-sheets and other proteins with the mostly-beta folds. For instance, the search with the SH3 domain from simulation #188 recovered 2001 predicted and experimentally solved SH3 structures. By contrast, a structurally similar but topologically distinct protein evolved in simulation #197 yielded only 92 hits, although it has a TM-score similarity of 0.68 with protein #188. Other examples of more complex evolved folds include the protein from simulation #194 resembling low-density lipoprotein receptor chaperone boca (pdbid: 3ofeB) (32), with TM-score 0.75 and RMSD 1.9 Å. Protein evolved in simulation #18 showed similarity with a fragment of V-ATPase subunit C (pdbid: 4dl0) with a TM-score of 0.73 and RMSD of 1.99 Å, although the respective sequences shared only 7% identity. Notably, several small proteins with simple folds, such as protein from simulation #100, which resembled a fragment of CRISPR-associated protein Cas5, in addition to the high structural similarity, also showed a relatively high sequence similarity (35 to 40%) although not detected by sequence search due to the small size of the similar regions (*SI Appendix*, Fig. S5). The complete list of Foldseek search results is provided in (*SI Appendix*, Table S2). Multiple novel folds, without significant similarity to any among known protein structures, also emerged as a result of the in silico evolution, in accord with the notion that protein language models can generalize beyond natural proteins and can be used to explore the protein universe (31).

Many details of protein evolution can be directly extracted from the simulations for further analysis, including a complete phylogenetic tree containing information about each mutation and all ancestral sequences. The examples in Fig. 2 *A* and *C* show the highest score for each generation and the full lineage of the protein with the highest score from the last generation. Although, at some points, this lineage overlapped with the best score of an intermediate generation, the trajectories of the ultimate winner and that of the best intermediate scores were different. Given that weak selection is a stochastic process in which a high fitness score increases the probability of survival but does not guarantee it, the protein with the highest score does not necessarily pass to the next generation. Conversely, variants with better scores often could not be found for several generations, and the fittest variant from the previous generation was repeatedly selected. As a result, the number of unique sequences in the lineage is always smaller than the number of generations. Detailed information about the changes in the protein sequence and the corresponding structural changes can be readily extracted from the lineage leading from the initial random sequence to the final sequence of the last generation with the best score as can be visualized at the level of secondary (Fig. 2 *B* and *D*) or tertiary structure (Movies S1 and S2).

More dramatic changes in the evolving protein structures often resulted from partial or complete sequence duplications, one of the primary modes of gene evolution (33, 34). Although duplications were rarely fixed in our simulations, they often lead to a major increase in fold complexity because after duplication, each of the two copies evolves separately. Once the new fold was established, changes in the structure were mostly incremental, affecting the loops or small secondary structure elements (*SI Appendix*, Fig. S6). Such a duplication event is well illustrated by the simulation presented in Fig. 2*C*, where a full duplication occurred after 400 generations, shaping a completely new fold with a beta-sheet and several alpha-helices. After duplication, the protein fold did not change its architecture during the rest of the simulation although there were minor changes in the loops that transformed into small alpha-helices, and an additional beta-strain was formed (Fig. 2*D* and Movie S2).

Duplications seem to be essential for the growth of evolving proteins, given that, in a broad variety of organisms, the rate of deletions is higher than the rate of insertions (35–37). Under this deletion bias, proteins would shrink with time, contradicting the fact that, at least, in many evolutionary lineages, protein complexity generally increases in the course of evolution (38–40). Furthermore, an increase in complexity should have been essential at the earliest stages of protein evolution. In our simulations, proteins were increasing in size most of the time due to selection, even when deletions were more frequent than insertions (*SI Appendix*, Fig. S7). Apparently, a lower insertion rate is compensated by the selection for the fitness score, which tends to be higher for larger proteins, and duplications provide an opportunity for a fast increase in fold complexity that is fixed if the duplicated structure is stable enough.

**Fig. 3.**
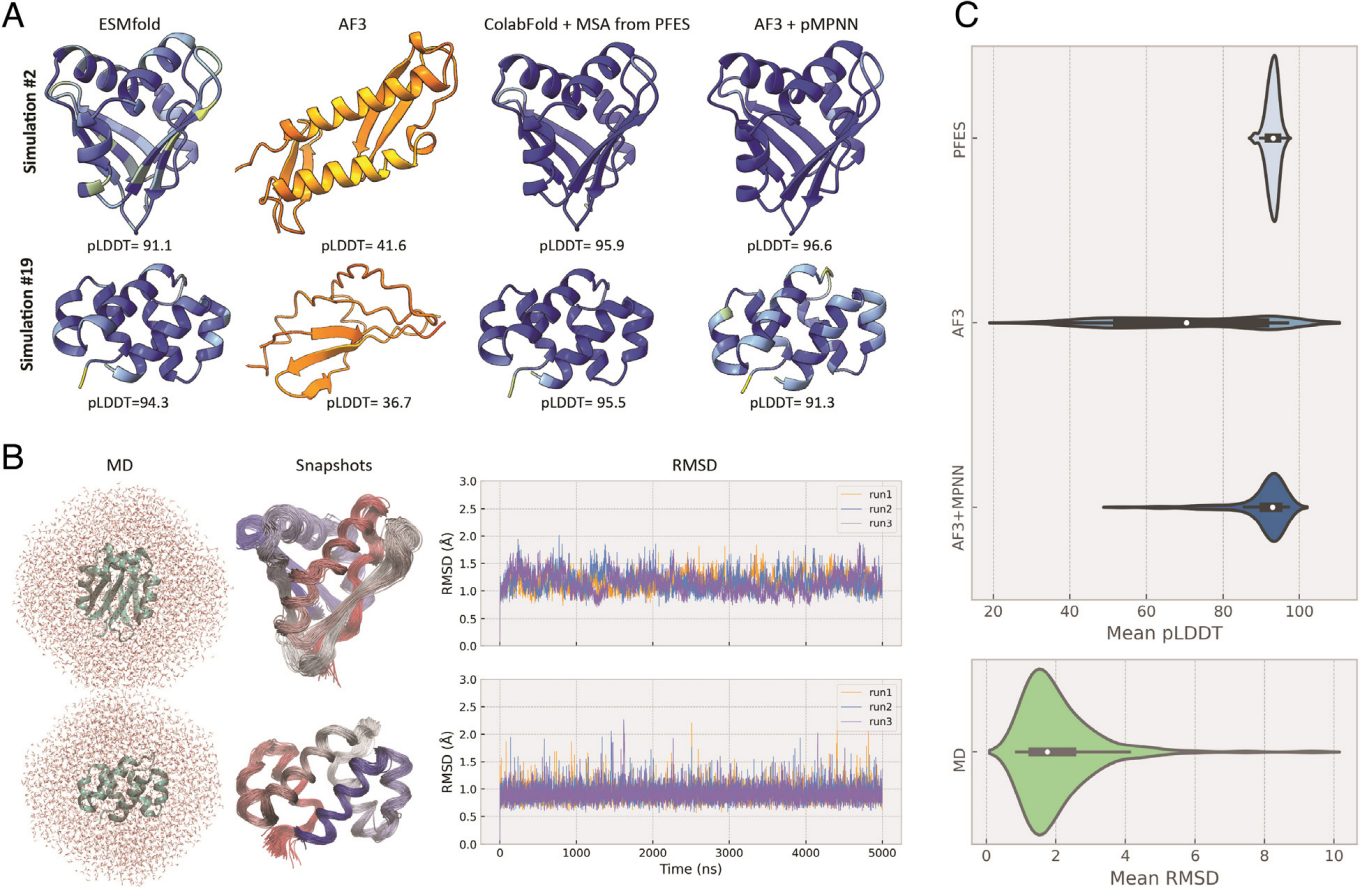
Stability of the evolved proteins. (*A*) Structures of proteins from simulations #2 and #19, predicted with ESMfold, AF3, ColabFold with an MSA extracted from PFES and AF3 with a sequence generated using inverse folding. (*B*) All-atom MD simulation with explicit water, superimposed snapshots from MD simulations colored from C (red) to N (blue) termini and their RMSD fluctuations over time, showing the stability of these proteins during the MD simulations. (*C*) Distribution of mean pLDDT from all simulations (n = 200) for structures produced by PFES (ESMfold), AF3, and AF3 with sequences generated by inverse folding, and distribution of mean RMSD from MD simulations, performed with the structures evolved in PFES.

Although sequence similarity searches provided limited or no information for the artificially evolved proteins, for 83 of the 200 evolved proteins, AlphaFold3 (AF3) nevertheless produced structural models with mean pLDDT > 80. The quality of the AlphaFold predictions strongly depends on the number of proteins homologous to the query that can be identified by sequence similarity search. Therefore, proteins with novel, unique folds from our simulation often could not be modeled with AF3, even when ESMfold predicted these structures with high confidence. For instance, the protein evolved in simulation #2 has a novel fold, which was not observed among the natural proteins. We ran a short simulation with strong selection to optimize this fold, which resulted in 72 mutations substituting 47% of residues in the initial sequence and improving the mean pLDDT from 83.5 to 91.1. The same optimized sequence predicted with AF3 had a mean pLDDT of 41.6 (Fig. 3*A*). However, when evolutionary information extracted from the simulations was provided as a multiple sequence alignment, it dramatically improved performance predicting a structure for the same sequence with a mean pLDDT of 95.9, that is, a confidence score even higher than that obtained initially with ESMfold (Fig. 3*A*). Protein from simulation #19 is another example with a novel fold, which after optimization had a mean pLDDT of 94.3 by ESMfold, whereas AF3 gave a pLDDT score of only 36.7. However, when information on residue conservation and coevolution from PFES was provided, the same structure as ESMfold was predicted, but with a dramatically improved mean pLDDT of 95.5.

To assess the robustness of the evolved folds, we used inverse folding with ProteinMPNN as a cross-validation approach because this method is not related to the ESMfold or AF3 (41). Using the structures that evolved in the simulations and inverse folding, we generated sequences that are supposed to adopt the same fold and for each protein chose the designed sequence with the best score to predict structure. The structure predictions obtained with AF3 for these redesigned sequences had much higher pLDDT score than the evolved structures themselves, and 182 of the 200 modeled proteins had pLDDT above 80 (Fig. 3 *A* and *C*). Most of these structures predicted with high confidence (177 of the 182) had the same fold as the original structures, with a TM-score greater than 0.5 and backbone RMSD less than 2.5 Å. This cross-validation demonstrates that three independent models for protein structure prediction and design (ESMfold, AF3, and ProteinMPNN) give a high probability that proteins evolved from random sequences in our simulations adopt stable folds.

In addition to the confidence scores obtained with ESMfold and AF3, in order to better assess the stability of the evolved proteins, we performed a series of all-atom molecular dynamics (MD) simulations for all artificially evolved proteins with three repeats in an explicit water environment for a more robust estimation of protein stability using physics-based simulations that do not depend on evolutionary or any other information except the predicted protein atom coordinates (*Methods*). We found that 163 of the 200 evolved proteins remained stable in all three MD simulations with a backbone RMSD below 2.5 Å and a SD of 1, even though 101 of these proteins had unique folds not detected among natural proteins (Fig. 3 *B* and *C* and *SI Appendix*, Fig. S8). The absence of dramatic deviations for the majority of the predicted structures during the simulation provides additional confidence that the predicted structures are stable.

### Number of Mutations Required for Fold Nucleation.

**Fig. 4.**
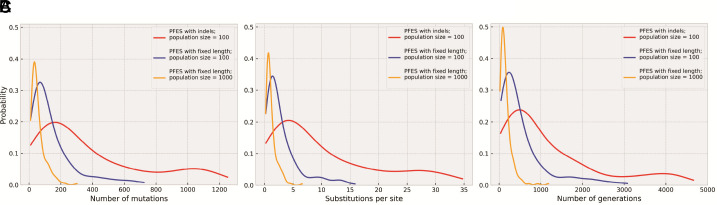
Number of mutations to fold nucleation estimated from simulations with fixed chain length or with indels. (*A*) Distribution of the number of mutations required for the evolution of a stable fold from a random sequence. (*B*) Number of mutations per site. (*C*) Number of generations to fold nucleation. The curves show probability density calculated with kernel density estimation (KDE). Detailed histograms are presented in *SI Appendix*, Fig. S9.

One crucial question that can be addressed with PFES is how many mutations are needed to evolve a stable protein fold from a peptide with a random sequence. Some small proteins have very simple structures, such as a beta-hairpin or an alpha-helix, but the evolution of larger proteins, usually containing more than 50 residues, strongly depends on the rates of duplication and insertion that are necessary to elongate the protein when the simulation starts from a short peptide. To circumvent the parameter search problem, we started simulations from 50 amino acids long polypeptides allowing only point mutations and single amino acid indels to maintain approximately the same length throughout the simulation. In this regime, we ran additional 200 independent simulations with a fixed population size of 100 members as in the previous runs until one of the peptides in the evolving population became stable, and as a condition for protein stability, pLDDT of 0.85 and pTM of 0.75 was selected. When the simulations reached the stopping conditions, we extracted lineages from each simulation to calculate the number of mutations necessary to evolve a stable protein from a random sequence. In this case, most of the evolved proteins had all-alpha folds such as HTH or helical bundles, and proteins with mixed folds containing both alpha helices and beta sheets, evolved more often than all-beta folds (*SI Appendix*, Fig. S3*A*). On average, it took 144.9 mutations to for a random polypeptide to evolve into a stable fold, that is, 2.97 mutations per site (Fig. 4 *A* and *B* and Table 1), considering that the length of the sequences did not change much during the simulations (*SI Appendix*, Fig. S3*B*). However, depending on the initial sequence and the evolutionary trajectory, the number of necessary mutations could vary dramatically. Whereas in some cases, only 10 mutations would suffice, in others, more than 500 mutations were needed to evolve a stable fold. It should be noted that this is the number of mutations only in the lineages that represented the direct path from the initial random sequence to the final protein. Many more mutations are needed to find this path: On average, it took 429 generations (or 42,931 mutations in total) to evolve a stable fold from a random sequence (Fig. 4*B*), that is, only 0.33% of the mutations were fixed. In simulations with a larger population size (1,000), on average, 56.77 mutations or 1.15 mutations per site were required (Fig. 4 *A* and *B*) to reach the same condition although more mutations occurred altogether and fewer of these mutations were fixed (Table 1). Moreover, in 38 of the 200 simulations with the population size of 1,000, less than 0.5 and in seven simulations less than 0.2 mutations per site were sufficient to evolve a stable fold (Fig. 4*B*).

**Table 1.** Key features of protein evolution simulated using PFES

	with indels	fixed length*	fixed length*
Population size	100	100	1,000
Initial peptide length	24	50	50
Average peptide length	56.7	48.9	49.5
Total number of mutations	102,693.88	42,931	118,670
Number of generations	1,026.94	429.31	118.67
Number of mutations	396.76	144.9	56.77
Fixation rate	0.00386	0.00338	0.00048
Mean sequence length	56.72	48.9	49.49
Mean number of mutations per site	10.53	2.97	1.15

Number of mutations/generations, average number of mutations/generations to evolve a stable fold in simulations; Total number of mutations, mean number of mutations that occurred in the course of evolution (population size × number of generations); Fixation rate, fraction of fixed mutations; Mean sequence length, the mean length of sequences at the end of the simulation; Mutations per site, the mean number of mutations per site calculated from each simulation. Simulations with indels included all types of mutations, in particular, duplications and indels (see Methods); simulations with fixed length included only single amino acid substitutions, insertions, and deletions.

We also calculated how many mutations and how many generations it took for an evolving protein to nucleate into a stable fold from the previously described simulations with larger indels and duplications allowed. However, because the evolving protein often changes the length, the accurate calculation of the number of mutations per site is problematic. In these simulations, most proteins adopted all-alpha folds (*SI Appendix*, Fig. S3*A*), and it took, on average, 397 mutations or 1,027 generations to evolve a stable protein from a pool of random 24-residue peptides (Fig. 4 *A* and *B*). Nevertheless, if we use the size of the final proteins (Table 1 and *SI Appendix*, Fig. S3*B*) there will be 10.53 mutations per site on average, but because the proteins were smaller at the beginning of the simulation and grew gradually via insertions and duplications, this is an overestimate of the required number of mutations.

### Evolution of Proteins with Interacting Partners.

**Fig. 5.**
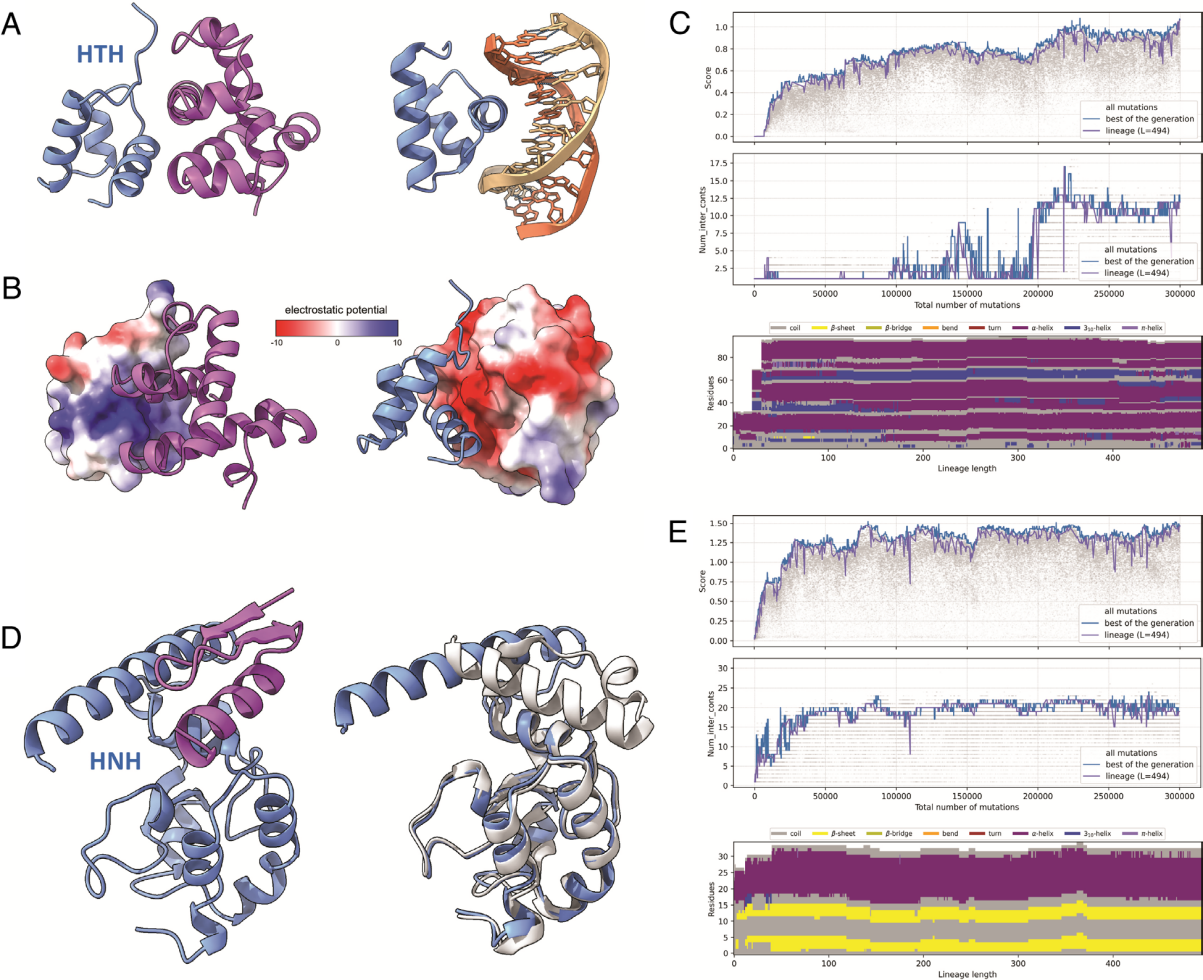
Evolution of a protein fold in complex with a binding partner. (*A*) The structure of a protein evolved from a random sequence (magenta) binding HTH of GcrA and the same HTH domain in a complex with dsDNA (PDBID: 5YIW). (*B*) Electrostatic surfaces of the HTH charged positive (*Left*), and the protein evolved in the simulation charged negative (*Right*). (*C*) Fitness score and the number of contacts between two chains plotted for all sequences (gray), the best sequences in each generation (blue), and the lineage of the best sequences from the last generation (violet). The effect of each mutation on the secondary structures is plotted for all mutations in the lineage. (*D*) The structure of an evolved protein in complex with HNH domain (blue). On the left is the conformation of the HNH domain in complex with the evolved protein (blue), superimposed with a free HNH domain that does not interact with other proteins (PDBID: 6O56, gray). The evolved protein is hidden for clarity. (*E*) Changes in the score, the number of contacts between two chains, and the secondary structures as described above.

Although protein fold stability is a necessary condition for most proteins to be functional, proteins do not exist in isolation but typically form a complex network of protein–protein interactions. Some of such interactions fulfill critical functions, which creates extra evolutionary pressure, in addition to the requirement of protein fold stability. To reach high fitness, a protein should often not only be stable but must also interact with specific, functionally important partners. We simulated a process whereby an evolving protein interacted with another protein that did not change through the simulation. The following example illustrates how random sequence evolves by interacting with a small DNA-binding domain with the HTH fold (bacterial cell cycle regulator GcrA) (42). The simulation of 3,000 generations was performed with stochastic selection, a population size of 100, and initial random sequences of 24 residues in length. The stable protein fold evolved during the first 250 generations and did not dramatically change thereafter (Fig. 5*A*). While evolving, the protein interacted with different parts of the HTH domain, and only after the 2,000th generation did the interaction stabilize as more contacts formed between the two chains (Fig. 5*C*). The C-terminal helix of the HTH carrying a positive charge was essential for the interaction with the negatively charged major grove of the DNA, and the evolved protein that contained a negatively charged pocket mimicked DNA (Fig. 5*A*), binding the HTH domain via electrostatic interactions.

We also ran a simulation with the same parameters as in the example above to reproduce the evolution of an anti-CRISPR protein (Acr) from a random sequence. Most of the numerous identified Acrs are small, fast-evolving proteins that interfere with different components of the bacterial and archaeal CRISPR-Cas adaptive immune system (43, 44). In this simulation, the protein was evolved from random sequences by interacting with the conserved HNH nuclease domain of the Cas9 protein that is essential for the target DNA cleavage and is targeted by several natural Acrs (45, 46). The random peptide evolved into a simple zinc finger fold with two beta-strands and an alpha-helix (beta-beta-alpha zinc finger, BBA) binding to a hydrophobic region of HNH consisting of residues 775 to 792, 802 to 818, and 887 to 893 (Fig. 5*D*). As in the previous cases, the major changes in the fold of the evolving protein occurred in the beginning of the simulation, whereas after ~500 generations, the fold and the interaction mode between the two proteins changed only marginally (Fig. 5*E*). The evolved protein was not charged as in the previous case, and the interaction between the two proteins was stabilized by the hydrophobic effect. The evolved protein bound the alpha-helix formed by residues 775 to 792 and changed its conformation upon binding (Fig. 5*B*). The binding of the evolved BBA protein did not directly obstruct the access to the catalytic site, as it does, for instance, in the case of AcrIIC1 (47), and did not block the PAM-recognition site like the DNA mimicking AcrIIC2, AcrIIA4, and AcrIC5 (48) but nevertheless, this interaction could lead to incorrect domain packing in Cas9, affecting its activity.

## Discussion

The emergence of stable, globular protein folds from random sequences is arguably the principal problem of protein evolution and one of the major challenges in the study of the origin of life. Once diverse globular folds evolved, the subsequent evolution of proteins throughout the 4 billion year history of life on Earth was a relatively straightforward and fairly well understood process. Successful attempts to computationally imitate protein evolution have been made previously, but these studies either focused on a particular part of the protein with amino acid substitutions that did not change the protein fold (49–51) or were limited to the exploration of simplified models in lattice space and reduced amino acid alphabets (7, 18). Here, in contrast, we simulated protein evolution with all-atom models, allowing us to observe how protein folds nucleated from random sequences and how the accumulation of amino acid substitutions led to large-scale conformational rearrangements, changing the general architecture of the fold. This critical difference that was made possible by the advent of powerful tools for protein structure prediction, such as AlphaFold and large language model based ESMfold, creates the opportunity to recapitulate protein fold evolution in detail not accessible previously.

The principal result of this work is that emergence of simple, stable, globular protein folds from random amino acid sequences is relatively easy and could occur quickly. Especially, under strong selection, many simulations readily produce such folds, some of which are closely similar to folds found in natural protein domains, such as HTH, beta hairpins, WW, or SH3. On average, we observed that nucleation of a stable fold required about 1.15 or 3 amino acid substitutions per site of the evolving amino acid sequence, depending on the population’s size and the evolutionary regime. Notably, in some simulations, as few as 0.2 substitutions per site sufficed to evolve a stable fold. It should be noted that even the lowest of these estimates were obtained for the low population size of 1,000 (simulations with larger population sizes would have been computationally prohibitive). This is a substantial but realistic amount of evolution. In different evolutionary lineages, the characteristic rates of protein evolution range between 0.2 and 1.5 amino acid substitutions per site per billion years (52). In a complementary study, for 57 highly conserved proteins, from 1.4 to 5.4 substitutions per site were estimated to have occurred in the about 4 billion years elapsed since the time of the Last Universal Common Ancestor (LUCA) (53). The time available for the emergence of protein folds at the early stage of life evolution was several hundred million years at best, under the latest robust estimates which suggest that well defined protein folds already existed earlier than 4 billion years ago (54). Furthermore, there are indications that the emergence of a diverse repertoire of protein folds antedated the formation of the full-fledged translation system, that is, apparently occurred at a stage of evolution directly following the primordial RNA World (39, 55). Thus, our conservative estimates of the number of amino acid substitutions required for a stable fold to evolve from a random polypeptide are within an order of magnitude of the evolutionary rates of some of the most highly conserved proteins with established, fully optimized folds that evolved under strong purifying selection since the time of the LUCA. It appears highly likely that evolution from random sequences at the primordial stages was much faster, driven by positive selection or, perhaps, more realistically, a combination of neutral evolution with positive selection, with purifying selection kicking in with the emergence of a minimally stable fold and functional constraints. Furthermore, reconstructions of evolutionary paths based on extant protein sequences disregard reverse mutations, leading to underestimates of the actual number of mutations. By contrast, in our simulations, the entire evolutionary trajectory is known, suggesting that the actual difference is even smaller.

The nature of the selection factors that drove protein evolution at early stages of evolution, before the advent of distinct protein folds, is an inherently difficult problem that currently may not admit a definitive solution. It should be noted that the stage of evolution we model in this work corresponds to the transition from an RNA-peptide world, in which the translation system, albeit primarily RNA-based one, and the genetic code have already evolved, but globular proteins have not. In the RNA-peptide world, peptides would serve as enhancers and stabilizers of ribozymes (17, 56, 57). The advent of bona fide proteins would greatly enhance these functions and would eventually lead to the replacement of most RNA catalysts with protein ones. For this transition to occur, stable, folded proteins and their interactions were essential, and furthermore, formation of globular folds could confer surface properties favoring interactions with other proteins, as demonstrated in some of our computational experiments, and with RNA. Thus, it appears most likely that fold stability and interactivity were key targets of selection at this stage. Notably, there are indications that the choice of the amino acids for incorporation into proteins, among the many prebiotically available ones, was dictated by the peptide foldability (58).

Certain structural motifs, such as alpha/beta hairpins, alpha-helical bundles, or beta sheets and sandwiches, that have been characterized as attractors in the protein structure space (59), recurrently emerged in many PFES simulations. By contrast, other attractor motifs, for example, beta-meanders, were observed rarely if at all. Further investigation of the structural features that are most likely to evolve from random sequences appears to be a promising direction to be pursued using PFES.

Taken together, our results suggest that evolution of globular protein folds from random sequences could be straightforward, requiring no unknown evolutionary processes, and in part, solve the enigma of rapid emergence of protein folds. Furthermore, the appearance, in many of the PFES runs, of simple folds closely similar to those found in natural proteins implies that evolutionary trajectories in the folding space are strongly constrained. The results of the evolutionary simulations with PFES are compatible with the “Biological Big Bang” model of protein evolution which shows how the protein universe could have evolved from a small number of primordial folds via duplication and diversification (60).

The major limitation of PFES is the limited accuracy of protein structure prediction methods. Although ESMfold provides a good tradeoff between speed and accuracy, it is questionable how biophysically realistic the predicted structures are, especially, structures with low confidence scores that often represent the intermediate states of protein fold evolution. Nevertheless, proteins in the native environments are not fixed constructs as experimental methods capture them but rather exist in a folding-unfolding equilibrium (61). The fraction of the unfolded protein is determined by the thermodynamic stability of the protein, that is, the folding free energy (62). The same fold predicted with ESMfold for different sequences can have different confidence scores, which might reflect the fraction of folded vs. unfolded proteins. Thus, a protein with a low confidence score is likely to maintain that fold transiently, whereas most of the time, such a protein is unfolded. Thus, sequences that possess only minimal functionality nevertheless could be subject to selection that will gradually stabilize the proteins, shifting the equilibrium toward a greater fraction of folded, stable proteins, with higher ESMfold scores. This is indeed the pattern of de novo protein evolution that we observed in PFES simulations.

Due to computational power limitations, we applied PFES to explore only a limited region of the protein evolution parameter space in a relatively small number of runs. However, PFES is a rich, flexible framework that, in principle, provides for a deep and broad computational study of protein evolution. With further development of PFES combined with improved prediction of protein structure and interactions with other proteins as well as nucleic acids, more realistic reproduction of primordial events at the origin of life will likely become feasible.

## Methods

### Protein Fold Evolution Simulation.

Protein fold evolution simulation (PFES) imitates the process of protein evolution by mutating a population of proteins, evaluating those mutations, and selecting the next generation for further evolution (Fig. 1). There are three predefined rates for mutation that can be additionally adjusted in an arbitrary way. In the presented simulations, the rate of single amino acid substitutions was uniformly distributed, and the probabilities used for nonsingle mutations are provided in (*SI Appendix*, Table S3). The size of the population (***N***) is defined at the beginning of the simulation and remains fixed. Each protein in the simulation mutates once, and the next generation is selected from the mixed population of original and mutated proteins of **2*N*** size. Strong or weak (stochastic) selection can be used in the simulations to determine which proteins will be transferred to the next generation. Strong selection (Eq. **1**) mode is deterministic, and only the best half of the mixed population forms the next generation.

[1] $$p_{i}=\begin{array}{cc} 1, & \text{if}\;s_{i}\left(t\right)>med\left(s_{1},s_{2}\cdots s_{2N}\right)\\ 0, & \text{otherwise} \end{array},$$

where $s_{i}$ is the fitness score of sequence *i*. Note that here, a given sequence may be selected at most once. However, the same sequence can appear in the population due to reoccurring mutations.

The probability in the stochastic selection used in these simulations was calculated assuming that $p_{i}$ is defined by relative fitness advantage:

[2] $$p_{i}=\frac{s_{i}}{\sum_{k=1}^{2N}s_{k}},$$

where $\sum_{k=1}^{2N}S_{k}$ is the sum of all scores. In this case, the same sequence can be selected multiple times depending on its score. The selection strength in the stochastic selection mode can be additionally regulated by a β factor as in the Gibbs distribution:

[3] $$p_{i}=\frac{e^{\beta s_{i}}}{\sum_{k=1}^{2N}e^{\beta S_{k}}}.$$

With β=[0,∞], where β=0 mean no selection that is $p_{i}=\frac{1}{2N}$, β=1, resembles stochastic selection as in Eq. **2**, and β>5, starts behaving as strong deterministic selection as is Eq. **1** (*SI Appendix*, Fig. S10).

The effect of each mutation is evaluated based on the structure predicted by the *esmfold_v1* pretrained model with one recycle (21). This is the bottleneck of the algorithm, and structures from each generation are split into several batches containing multiple sequences, which are predicted together for optimal usage of the GPU. While the next batch is being predicted, the algorithm calculates scores for the previous batch using a Central Processing Unit (CPU), thus running two processes in parallel to optimize the performance. The final fitness score is a multiplicative function that includes pLDDT, pTM, contact density (CD), and constraints if they are applied Eq. **4**.

[4] $$s_{i}=\text{pLDDT × pTM × CD × (ipLDDT × iCD) × (}\text{constr}\text{)}.$$

The first two terms (the average pLDDT across all residues and pTM), obtained directly from the ESMfold prediction, show the general prediction quality. CD is calculated as the number of contacts normalized by the protein length *l* (Eq. **5**). Contacts are calculated between C-beta atoms if Euclidean distance between those are closer than 6 Å, residues are located five positions apart in the sequence (*d*1 and *d*2 in Eq. **5**) and pLDDT for both residues is more than 50, so contacts in the alpha-helices or low pLDDT regions are not counted.

[5] $$\begin{array}{c} \mathrm{CD}\;=\;\frac{1}{l}\sum c\left(i,\;j\right)+1;\;c\left(i,\;j\right)\;=\;\begin{array}{cc} 1; & \mathrm{if}\;d1\left(i,\;j\right)\;<\;0.6\;A\;\&\;d2\left(i,\;j\right)\;\mathrm{is}\;>\;5\;\mathrm{res}\;\&\;\mathrm{pLDDT}\;>\;50\\ 0; & \mathrm{otherwise} \end{array}. \end{array}$$

For dimers, the score includes two additional components: ipLDDT, which is pLDDT on the interface of interacting proteins, that includes residues between chains where C-beta atoms are closer than 6 Å, and *iCD,* which is the number of contacts between chains calculated in the same way and normalized by the length of the evolving protein.

Additional correction in the score can be introduced through constraints (*constr* in Eq. **4**) for protein or secondary structure length (P_L_ and P_α_, P_β_), which are functions with values from 0 to 1 depending on protein or secondary structure lengths (Eqs. **6**–**8**). For instance, if protein length is within the allowed range, the score is multiplied by 1 and does not change, but if it becomes equal to the $L_{0}$, the score is multiplied by 0.5.

[6] $$P_{L}=1-\frac{1}{1+e^{C\left(L0-Lp\right)}};\;C=0.2;L_{0}=250,$$

[7] $$P_{\alpha }=1-\frac{1}{1+e^{C\left(L0-Lh\right)}};\;C=0.5;L_{0}=30,$$

[8] $$P_{\beta }=1-\frac{1}{1+e^{C\left(L0-Lh\right)}};\;C=0.5;L_{0}=12.$$

Secondary structure elements are identified from the predicted structures using PSIQUE (P(63)). In the simulations in this work, the secondary structure constraints $L_{0}$ for beta-sheets was 12 and for alpha-helices 30.

Three sets of simulations were run with 200 repeats in each. The first set consisted of simulations that included all possible mutations mentioned above and was performed for 4,000 generations with stochastic selection and 1,000 generations with strong selection at the end. The population’s size was 100 and the initial population was started from a random sequence with a length of 24 amino acids. The second set of simulations was performed with constrained length using only point mutations and single amino acid indels. The initial population was started from a random peptide with a length of 50 amino acids, the population size was 100. The third set of simulations was performed under the same conditions, but with a population size of 1000. The second and third sets of simulations were run with stochastic selection until one of the peptides in the generation reached pLDDT of 85 and pTM of 0.75. PFES performance scales quadratically with the total length of the sequences in the evolving protein population.

### Structure Similarity Search and Clustering.

GTalign was used for clustering structures with a TM-score of 0.5 and coverage of 0.7 with speed mode 0 (64). MMseqs2 with a sequence identity of 30% and bilateral coverage of 0.7 was used for sequence clustering (65). Foldseek was used for structure-based search in AFDB/UniProt50, MGnify-ESM30, and PDB with alignment type 3Di+AA and a sensitivity of 9.5 (30). Hits were filtered with probability >0.95 and query coverage 0.8. PSI-BLAST with word size 2 was used for search in the NR database (66).

### MD Simulations.

MD simulations were performed using Amber20 software and ff19SB protein forcefield (67, 68). Proteins were solvated with the TIP3P water model and K^+^/Cl^−^ ions in truncated octahedral boxes with 10 Å buffer between protein and box. The systems were minimized and equilibrated gradually, releasing restraints, and the final simulations were performed at a constant temperature of 298.15 K and pressure of 1 bar using the Langevin thermostat with a collision frequency of 2 ps^−1^ and Monte Carlo barostat, respectively. The Particle Mesh Ewald (PME) approach with a short nonbonded cutoff of 10 Å was used for treating long-range electrostatics. For the final simulations, hydrogen mass repartitioning and 4 fs integration timestep was used. MD simulations lasting 500 ns were repeated three times for each protein, except for proteins from simulations #2 and #19 which simulations were performed for 5,000 ns. RMSD for each trajectory was calculated for backbone atoms with *cpptraj* using the equilibrated structure as a ref. 69.

### Protein Structure Prediction.

Structures of artificially evolved proteins were predicted using AF3 with default parameters which was installed locally on HPC Biowulf (19). ColabFold v1.5.5 was used to predict structures with custom MSAs, which were extracted from PFES (70).

### Inverse Folding.

ProteinMPNN with model v_48_020 (41) was used for inverse folding. Sequences corresponding to the predetermined protein backbone were generated with a sampling temperature at 0.1. For each protein 100 sequences were generated from which the sequence with the best score was used to predict structure with AF3.

### Molecular Graphics.

For protein structure visualization, ChimeraX and VMD (Visual Molecular Dynamics) were used (71, 72). PFES lineage trajectories showing changes in the protein structure can be visualized in VMD (72).

## Supplementary Material

Appendix 01 (PDF)

Dataset S01 (CSV)

Movie S1.

Movie S2.

## Data Availability

PFES code is available at github.com/sahakyanhk/pfes (73). The simulation files are available at zenodo.org/records/15588153 (74).

## References

[1] B. Alberts , Molecular Biology of the Cell (W.W. Norton & Co, ed. 7, 2022).

[2] A. V. Finkelstein, O. B. Ptitsyn, Protein Physics: A Course of Lectures (Academic Press, NY, 2002).

[3] C. Chothia, Proteins. One thousand families for the molecular biologist. Nature **357**, 543–544 (1992). 1608464 10.1038/357543a0

[4] E. V. Koonin, Y. I. Wolf, G. P. Karev, The structure of the protein universe and genome evolution. Nature **420**, 218–223 (2002). 12432406 10.1038/nature01256

[5] L. A. Mirny, V. I. Abkevich, E. I. Shakhnovich, How evolution makes proteins fold quickly. Proc. Natl. Acad. Sci. U.S.A. **95**, 4976–4981 (1998). 9560213 10.1073/pnas.95.9.4976 PMC20198

[6] K. A. Dill , Principles of protein folding–A perspective from simple exact models. Protein Sci. **4**, 561–602 (1995). 7613459 10.1002/pro.5560040401 PMC2143098

[7] A. E. Lobkovsky, Y. I. Wolf, E. V. Koonin, Universal distribution of protein evolution rates as a consequence of protein folding physics. Proc. Natl. Acad. Sci. U.S.A. **107**, 2983–2988 (2010). 20133769 10.1073/pnas.0910445107 PMC2840281

[8] J. Soding, A. N. Lupas, More than the sum of their parts: On the evolution of proteins from peptides. Bioessays **25**, 837–846 (2003). 12938173 10.1002/bies.10321

[9] V. Alva, J. Soding, A. N. Lupas, A vocabulary of ancient peptides at the origin of folded proteins. Elife **4**, e09410 (2015). 26653858 10.7554/eLife.09410 PMC4739770

[10] A. N. Lupas, C. P. Ponting, R. B. Russell, On the evolution of protein folds: Are similar motifs in different protein folds the result of convergence, insertion, or relics of an ancient peptide world? J. Struct. Biol. **134**, 191–203 (2001). 11551179 10.1006/jsbi.2001.4393

[11] S. Nepomnyachiy, N. Ben-Tal, R. Kolodny, Complex evolutionary footprints revealed in an analysis of reused protein segments of diverse lengths. Proc. Natl. Acad. Sci. U.S.A. **114**, 11703–11708 (2017). 29078314 10.1073/pnas.1707642114 PMC5676897

[12] R. Kolodny, S. Nepomnyachiy, D. S. Tawfik, N. Ben-Tal, Bridging themes: Short protein segments found in different architectures. Mol. Biol. Evol. **38**, 2191–2208 (2021). 33502503 10.1093/molbev/msab017 PMC8136508

[13] A. N. Lupas, V. Alva, Ribosomal proteins as documents of the transition from unstructured (poly)peptides to folded proteins. J. Struct. Biol. **198**, 74–81 (2017). 28454764 10.1016/j.jsb.2017.04.007

[14] V. Alva, A. N. Lupas, From ancestral peptides to designed proteins. Curr. Opin. Struct. Biol. **48**, 103–109 (2018). 29195087 10.1016/j.sbi.2017.11.006

[15] L. I. Gutierrez-Rus , Protection of catalytic cofactors by polypeptides as a driver for the emergence of primordial enzymes. Mol. Biol. Evol., 10.1093/molbev/msad126 (2023). PMC10279418 37235753

[16] S. D. Fried, K. Fujishima, M. Makarov, I. Cherepashuk, K. Hlouchova, Peptides before and during the nucleotide world: An origins story emphasizing cooperation between proteins and nucleic acids. J. R. Soc. Interface **19**, 20210641 (2022). 35135297 10.1098/rsif.2021.0641 PMC8833103

[17] Y. I. Wolf, E. V. Koonin, On the origin of the translation system and the genetic code in the RNA world by means of natural selection, exaptation, and subfunctionalization. Biol. Direct **2**, 14 (2007). 17540026 10.1186/1745-6150-2-14 PMC1894784

[18] E. Guseva, R. N. Zuckermann, K. A. Dill, Foldamer hypothesis for the growth and sequence differentiation of prebiotic polymers. Proc. Natl. Acad. Sci. U.S.A. **114**, E7460–E7468 (2017). 28831002 10.1073/pnas.1620179114 PMC5594640

[19] J. Jumper , Highly accurate protein structure prediction with AlphaFold. Nature **596**, 583–589 (2021). 34265844 10.1038/s41586-021-03819-2 PMC8371605

[20] J. Abramson , Accurate structure prediction of biomolecular interactions with AlphaFold 3. Nature **630**, 493–500 (2024). 38718835 10.1038/s41586-024-07487-w PMC11168924

[21] Z. Lin , Evolutionary-scale prediction of atomic-level protein structure with a language model. Science **379**, 1123–1130 (2023). 36927031 10.1126/science.ade2574

[22] R. Krishna , Generalized biomolecular modeling and design with RoseTTAFold All-Atom. Science **384**, eadl2528 (2024).38452047 10.1126/science.adl2528

[23] S. Henikoff, J. G. Henikoff, Amino acid substitution matrices from protein blocks. Proc. Natl. Acad. Sci. U.S.A. **89**, 10915–10919 (1992). 1438297 10.1073/pnas.89.22.10915 PMC50453

[24] K. Tomii, M. Kanehisa, Analysis of amino acid indices and mutation matrices for sequence comparison and structure prediction of proteins. Protein Eng. **9**, 27–36 (1996). 9053899 10.1093/protein/9.1.27

[25] K. A. Geiler-Samerotte , Misfolded proteins impose a dosage-dependent fitness cost and trigger a cytosolic unfolded protein response in yeast. Proc. Natl. Acad. Sci. U.S.A. **108**, 680–685 (2011). 21187411 10.1073/pnas.1017570108 PMC3021021

[26] D. A. Drummond, C. O. Wilke, The evolutionary consequences of erroneous protein synthesis. Nat. Rev. Genet. **10**, 715–724 (2009). 19763154 10.1038/nrg2662 PMC2764353

[27] V. N. Uversky, On the roles of protein intrinsic disorder in the origin of life and evolution. Life (Basel), 10.3390/life14101307 (2024). PMC11509291 39459607

[28] S. Gavrilets, Fitness Landscapes and the Origin of Species (Princeton University Press, Princeton, 2004).

[29] C. Alvarez-Carreno, P. I. Penev, A. S. Petrov, L. D. Williams, Fold evolution before LUCA: Common ancestry of SH3 domains and OB domains. Mol. Biol. Evol. **38**, 5134–5143 (2021). 34383917 10.1093/molbev/msab240 PMC8557408

[30] M. van Kempen , Fast and accurate protein structure search with Foldseek. Nat. Biotechnol. **42**, 243–246 (2024). 37156916 10.1038/s41587-023-01773-0 PMC10869269

[31] R. Verkuil , Language models generalize beyond natural proteins. bioRxiv [Preprint] (2022). https://www.biorxiv.org/content/10.1101/2022.12.21.521521v1 (Accessed 6 March 2025).

[32] M. N. Collins, W. A. Hendrickson, Structural characterization of the Boca/Mesd maturation factors for LDL-receptor-type beta propeller domains. Structure **19**, 324–336 (2011). 21397184 10.1016/j.str.2010.11.017 PMC3099344

[33] S. Ohno, Evolution by Gene Duplication (Springer-Verlag, Berlin-Heidelberg-New York, 1970).

[34] M. Lynch, J. S. Conery, The evolutionary fate and consequences of duplicate genes. Science **290**, 1151–1155 (2000). 11073452 10.1126/science.290.5494.1151

[35] E. V. Leushkin, G. A. Bazykin, A. S. Kondrashov, Strong mutational bias toward deletions in the Drosophila melanogaster genome is compensated by selection. Genome Biol. Evol. **5**, 514–524 (2013). 23395983 10.1093/gbe/evt021 PMC3622295

[36] H. J. Barton, K. Zeng, New methods for inferring the distribution of fitness effects for INDELs and SNPs. Mol. Biol. Evol. **35**, 1536–1546 (2018). 29635416 10.1093/molbev/msy054 PMC5967470

[37] C. H. Kuo, H. Ochman, Deletional bias across the three domains of life. Genome Biol. Evol. **1**, 145–152 (2009). 20333185 10.1093/gbe/evp016 PMC2817411

[38] E. V. Koonin, L. Aravind, A. S. Kondrashov, The impact of comparative genomics on our understanding of evolution. Cell **101**, 573–576 (2000). 10892642 10.1016/s0092-8674(00)80867-3

[39] L. Aravind, R. Mazumder, S. Vasudevan, E. V. Koonin, Trends in protein evolution inferred from sequence and structure analysis. Curr. Opin. Struct. Biol. **12**, 392–399 (2002). 12127460 10.1016/s0959-440x(02)00334-2

[40] A. Kauko, K. Lehto, Eukaryote specific folds: Part of the whole. Proteins **86**, 868–881 (2018). 29675831 10.1002/prot.25517

[41] J. Dauparas , Robust deep learning-based protein sequence design using ProteinMPNN. Science **378**, 49–56 (2022). 36108050 10.1126/science.add2187 PMC9997061

[42] X. Wu , Structural insights into the unique mechanism of transcription activation by Caulobacter crescentus GcrA. Nucleic Acids Res. **46**, 3245–3256 (2018). 29514271 10.1093/nar/gky161 PMC5887438

[43] T. Wiegand, S. Karambelkar, J. Bondy-Denomy, B. Wiedenheft, Structures and strategies of anti-CRISPR-mediated immune suppression. Annu. Rev. Microbiol. **74**, 21–37 (2020). 32503371 10.1146/annurev-micro-020518-120107 PMC7712631

[44] Y. Li, J. Bondy-Denomy, Anti-CRISPRs go viral: The infection biology of CRISPR-Cas inhibitors. Cell Host Microbe **29**, 704–714 (2021). 33444542 10.1016/j.chom.2020.12.007 PMC8122014

[45] B. A. Osuna , Listeria phages induce Cas9 degradation to protect lysogenic genomes. Cell Host Microbe **28**, 31–40.e9 (2020). 32325050 10.1016/j.chom.2020.04.001 PMC7351598

[46] Y. Kim , Anti-CRISPR AcrIIC3 discriminates between Cas9 orthologs via targeting the variable surface of the HNH nuclease domain. FEBS J. **286**, 4661–4674 (2019). 31389128 10.1111/febs.15037

[47] Y. Zhu, S. Yin, Z. Li, Mechanism of inhibition of CRISPR-Cas9 by anti-CRISPR protein AcrIIC1. Biochem. Biophys. Res. Commun. **654**, 34–39 (2023). 36878037 10.1016/j.bbrc.2023.02.065

[48] S. Hwang , Anti-CRISPR protein AcrIIC5 inhibits CRISPR-Cas9 by occupying the target DNA binding pocket. J. Mol. Biol. **435**, 167991 (2023). 36736884 10.1016/j.jmb.2023.167991

[49] C. Norn, I. Andre, Atomistic simulation of protein evolution reveals sequence covariation and time-dependent fluctuations of site-specific substitution rates. PLoS Comput. Biol. **19**, e1010262 (2023). 36961827 10.1371/journal.pcbi.1010262 PMC10075473

[50] Q. Jiang, A. I. Teufel, E. L. Jackson, C. O. Wilke, Beyond thermodynamic constraints: Evolutionary sampling generates realistic protein sequence variation. Genetics **208**, 1387–1395 (2018). 29382650 10.1534/genetics.118.300699 PMC5887137

[51] S. A. Raven, B. Payne, M. Bruce, A. Filipovska, O. Rackham, In silico evolution of nucleic acid-binding proteins from a nonfunctional scaffold. Nat. Chem. Biol. **18**, 403–411 (2022). 35210620 10.1038/s41589-022-00967-y

[52] I. K. Jordan , A universal trend of amino acid gain and loss in protein evolution. Nature **433**, 633–638 (2005). 15660107 10.1038/nature03306

[53] E. R. R. Moody , The nature of the last universal common ancestor and its impact on the early Earth system. Nat. Ecol. Evol. **8**, 1654–1666 (2024). 38997462 10.1038/s41559-024-02461-1 PMC11383801

[54] T. A. Mahendrarajah , ATP synthase evolution on a cross-braced dated tree of life. Nat. Commun. **14**, 7456 (2023). 37978174 10.1038/s41467-023-42924-w PMC10656485

[55] L. Aravind, V. Anantharaman, E. V. Koonin, Monophyly of class I aminoacyl tRNA synthetase, USPA, ETFP, photolyase, and PP-ATPase nucleotide-binding domains: Implications for protein evolution in the RNA. Proteins **48**, 1–14 (2002). 12012333 10.1002/prot.10064

[56] M. Di Giulio, On the RNA world: Evidence in favor of an early ribonucleopeptide world. J. Mol. Evol. **45**, 571–578 (1997). 9419234 10.1007/pl00006261

[57] F. Muller , A prebiotically plausible scenario of an RNA-peptide world. Nature **605**, 279–284 (2022). 35546190 10.1038/s41586-022-04676-3 PMC9095488

[58] M. Makarov , Early selection of the amino acid alphabet was adaptively shaped by biophysical constraints of foldability. J. Am. Chem. Soc. **145**, 5320–5329 (2023). 36826345 10.1021/jacs.2c12987 PMC10017022

[59] L. Holm, C. Sander, Mapping the protein universe. Science **273**, 595–603 (1996). 8662544 10.1126/science.273.5275.595

[60] N. V. Dokholyan, B. Shakhnovich, E. I. Shakhnovich, Expanding protein universe and its origin from the biological Big Bang. Proc. Natl. Acad. Sci. U.S.A. **99**, 14132–14136 (2002). 12384571 10.1073/pnas.202497999 PMC137849

[61] A. V. Finkelstein, N. S. Bogatyreva, D. N. Ivankov, S. O. Garbuzynskiy, Protein folding problem: Enigma, paradox, solution. Biophys. Rev. **14**, 1255–1272 (2022). 36659994 10.1007/s12551-022-01000-1 PMC9842845

[62] K. Lindorff-Larsen, K. Teilum, Linking thermodynamics and measurements of protein stability. Protein Eng. Des. Sel., 10.1093/protein/gzab002 (2021). 33724431

[63] F. Adasme-Carreno, J. Caballero, J. Ireta, PSIQUE: Protein secondary structure identification on the basis of quaternions and electronic structure calculations. J. Chem. Inf. Model **61**, 1789–1800 (2021). 33769809 10.1021/acs.jcim.0c01343

[64] M. Margelevicius, GTalign: Spatial index-driven protein structure alignment, superposition, and search. Nat. Commun. **15**, 7305 (2024). 39181863 10.1038/s41467-024-51669-z PMC11344802

[65] M. Steinegger, J. Soding, MMseqs2 enables sensitive protein sequence searching for the analysis of massive data sets. Nat. Biotechnol. **35**, 1026–1028 (2017). 29035372 10.1038/nbt.3988

[66] S. F. Altschul , Gapped BLAST and PSI-BLAST: A new generation of protein database search programs. Nucleic Acids Res. **25**, 3389–3402 (1997). 9254694 10.1093/nar/25.17.3389 PMC146917

[67] R. Salomon-Ferrer, A. W. Gotz, D. Poole, S. Le Grand, R. C. Walker, Routine microsecond molecular dynamics simulations with AMBER on GPUs. 2. Explicit solvent particle mesh Ewald. J. Chem. Theory Comput. **9**, 3878–3888 (2013). 26592383 10.1021/ct400314y

[68] A. M. Fischer , The role of force fields and water models in protein folding and unfolding dynamics. J. Chem. Theory Comput. **20**, 2321–2333 (2024). 38373307 10.1021/acs.jctc.3c01106 PMC10938642

[69] D. R. Roe, T. E. Cheatham III, Parallelization of CPPTRAJ enables large scale analysis of molecular dynamics trajectory data. J. Comput Chem. **39**, 2110–2117 (2018). 30368859 10.1002/jcc.25382 PMC7313716

[70] M. Mirdita , ColabFold: Making protein folding accessible to all. Nat. Methods **19**, 679–682 (2022). 35637307 10.1038/s41592-022-01488-1 PMC9184281

[71] E. F. Pettersen , UCSF ChimeraX: Structure visualization for researchers, educators, and developers. Protein Sci. **30**, 70–82 (2021). 32881101 10.1002/pro.3943 PMC7737788

[72] W. Humphrey, A. Dalke, K. Schulten, VMD: Visual molecular dynamics. J. Mol. Graph **14**, 33–38, 27–38 (1996). 8744570 10.1016/0263-7855(96)00018-5

[73] H. Sahakyan, S. G. Babajanyan, Y. I. Wolf, E. V. Koonin, PFES: protein fold evolution simulation. GitHub. https://github.com/sahakyanhk/pfes. Accessed 3 June 2025.

[74] H. Sahakyan, S. G. Babajanyan, Y. I. Wolf, E. V. Koonin In silico evolution of globular protein folds from random sequences (extended data). Zenodo. 10.5281/zenodo.15588153. Accessed 3 June 2025. PMC12260532 40587803

