# Supplementary material for: In silico evolution of globular protein folds from random sequences
Source: Proc Natl Acad Sci U S A. 2025 Jun 30;122(27):e2509015122. doi: 10.1073/pnas.2509015122 (PMC12260532; doi:10.1073/pnas.2509015122)
Supplement: Supplementary file 1 — Appendix 01 (PDF) [file pnas.2509015122.sapp.pdf]

Supporting Information Appendix

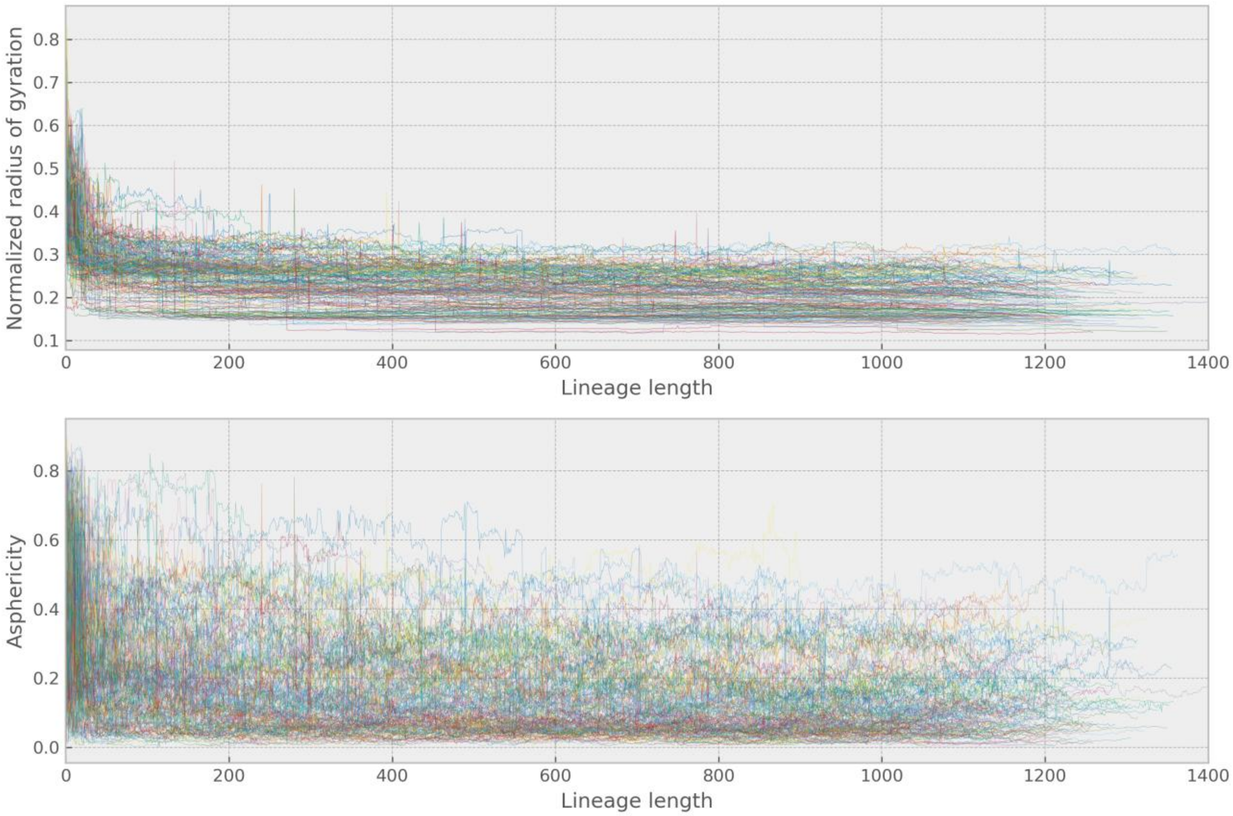

Figure S1. The radius of gyration (R_g) normalized by protein length calculated for proteins in simulations with insertions (n=200), shows compactization of evolving peptides over time. Asphericity, a length-independent metric scaling from 0 (ideal sphere) to 1 (ideal line), also indicates the compactization of proteins during artificial evolution.

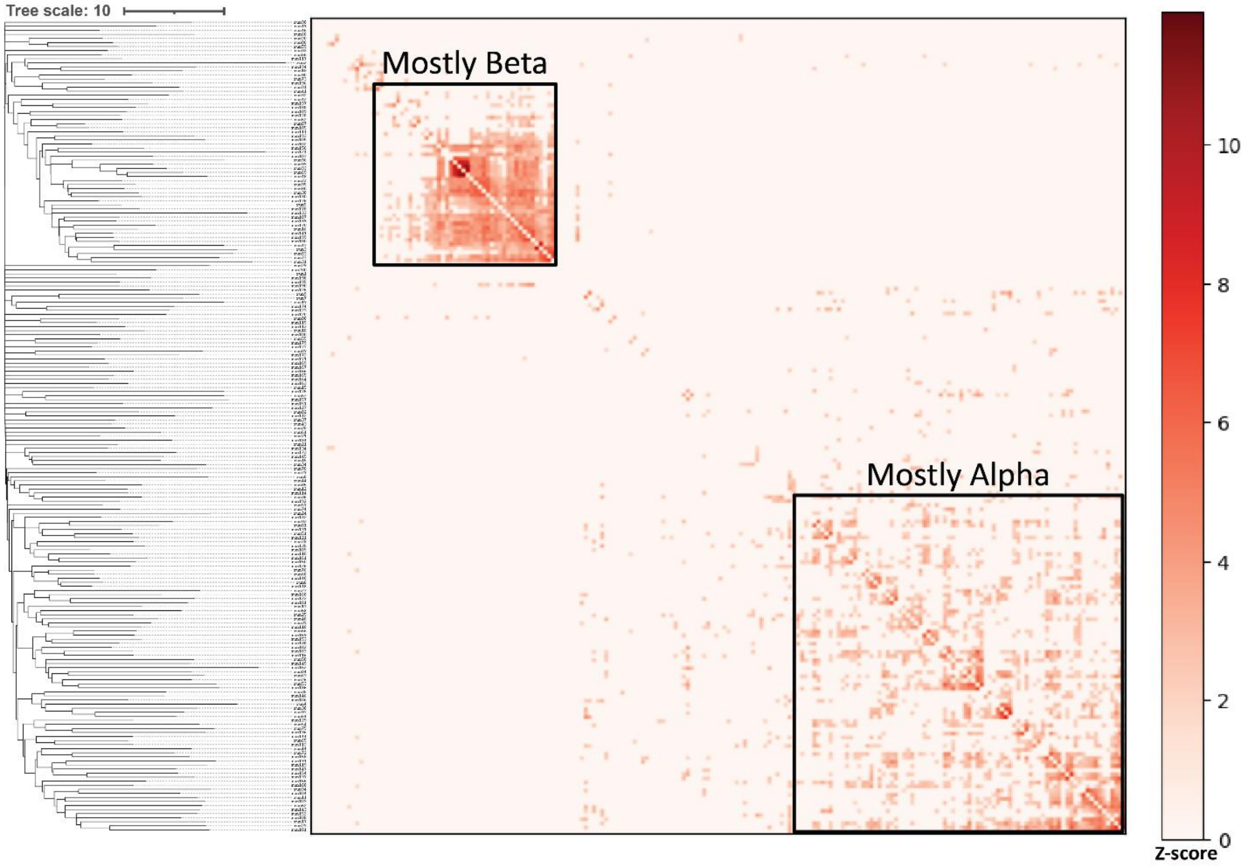

Figure S2. All-versus-all comparison of the evolved structures using Dali. A high Z-score indicates higher similarity between the compared structures. The top-left cluster represents structures that contain a similar beta-sheet, and the scattered cluster on the bottom-left represents structures containing similar alpha-helices.

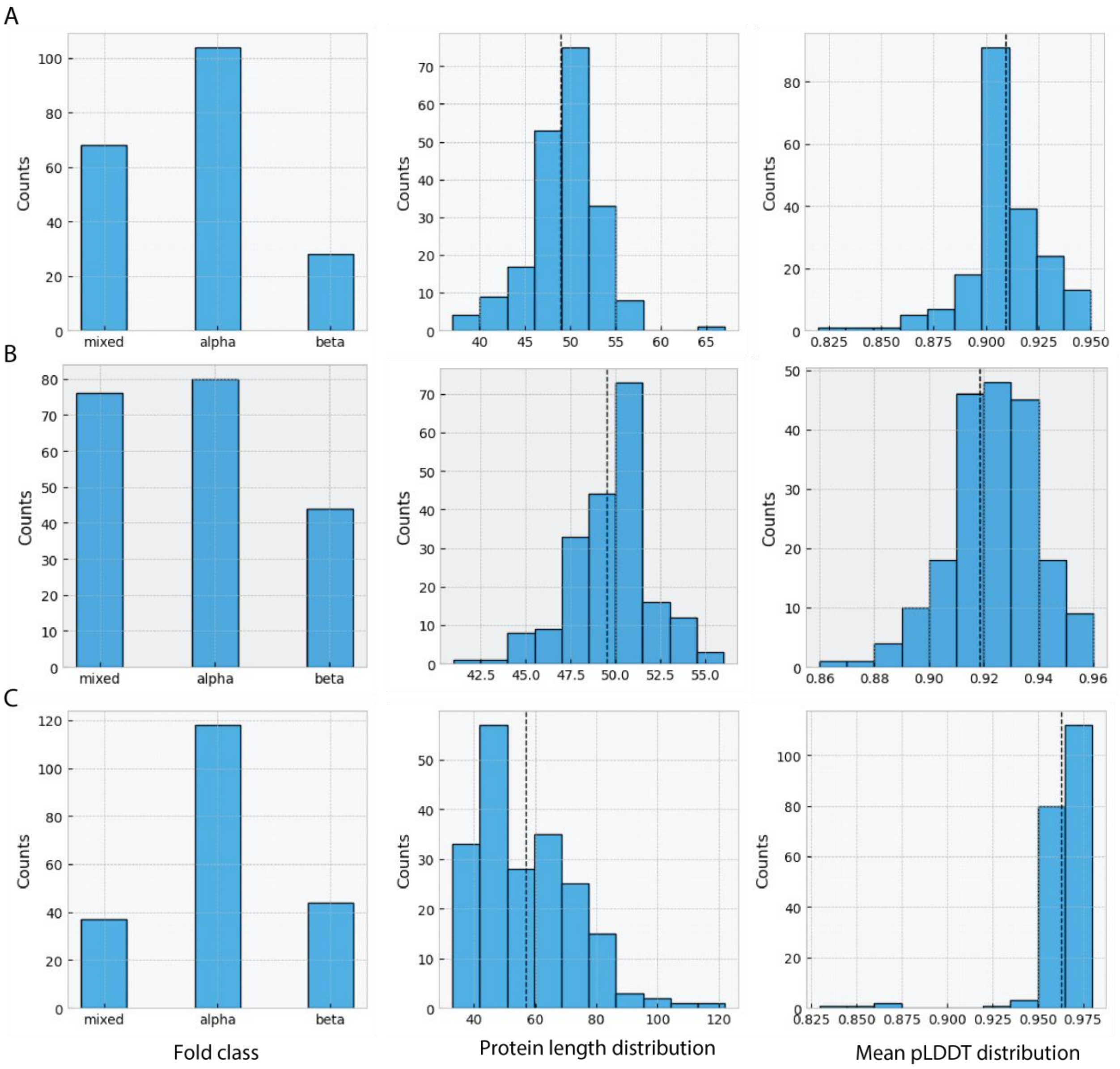

Figure S3. Protein fold class, length, and mean pLDDT distributions in PFES. The dashed line shows the mean of the distribution. (A) Simulations with indels and population size of 100, which include full or partial duplication and insertions of different lengths, started from random sequences containing 24 residues. (B) Simulations with constrained protein length and a population size of 100 were initiated with random sequences containing 50 residues, and only single amino acid indels and substitutions were permitted. (C) The same simulations with a fixed length but a population size of 1000. See Table 1 for details.

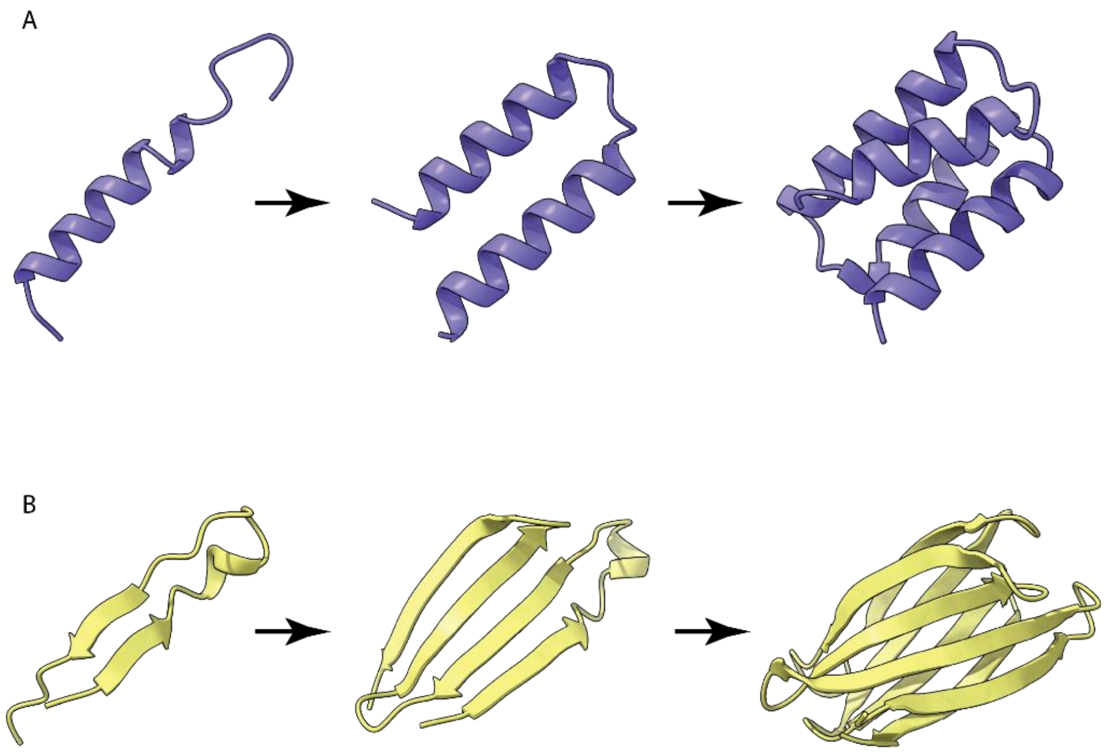

Figure S4. Recurrent patterns of duplications leading to the formation of (A) alpha-helical bundles and (B) beta-sandwiches.

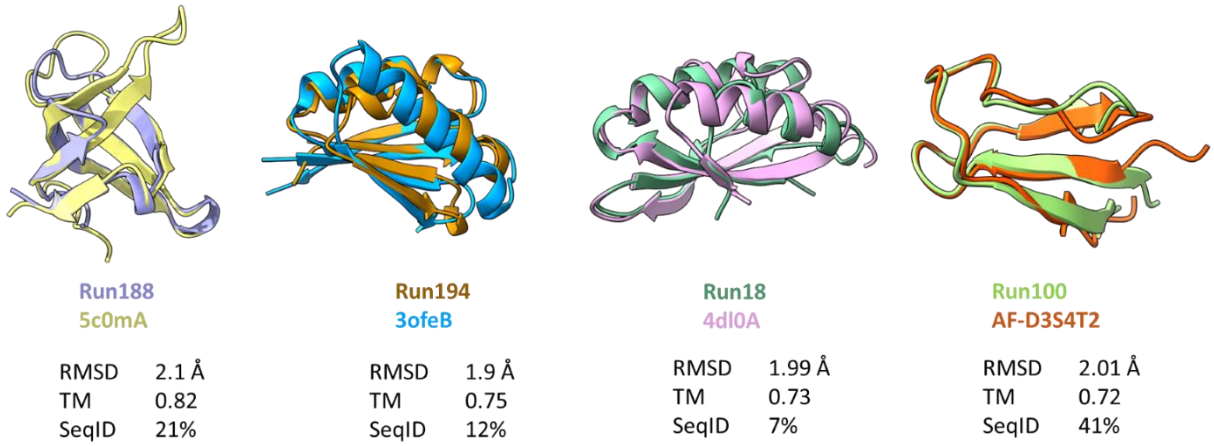

Figure S5. Proteins from simulated evolution are structurally similar to natural proteins from the PDB and AFDB databases. Under each superposition, their corresponding RMSD, TM-score, and sequence identity are indicated.

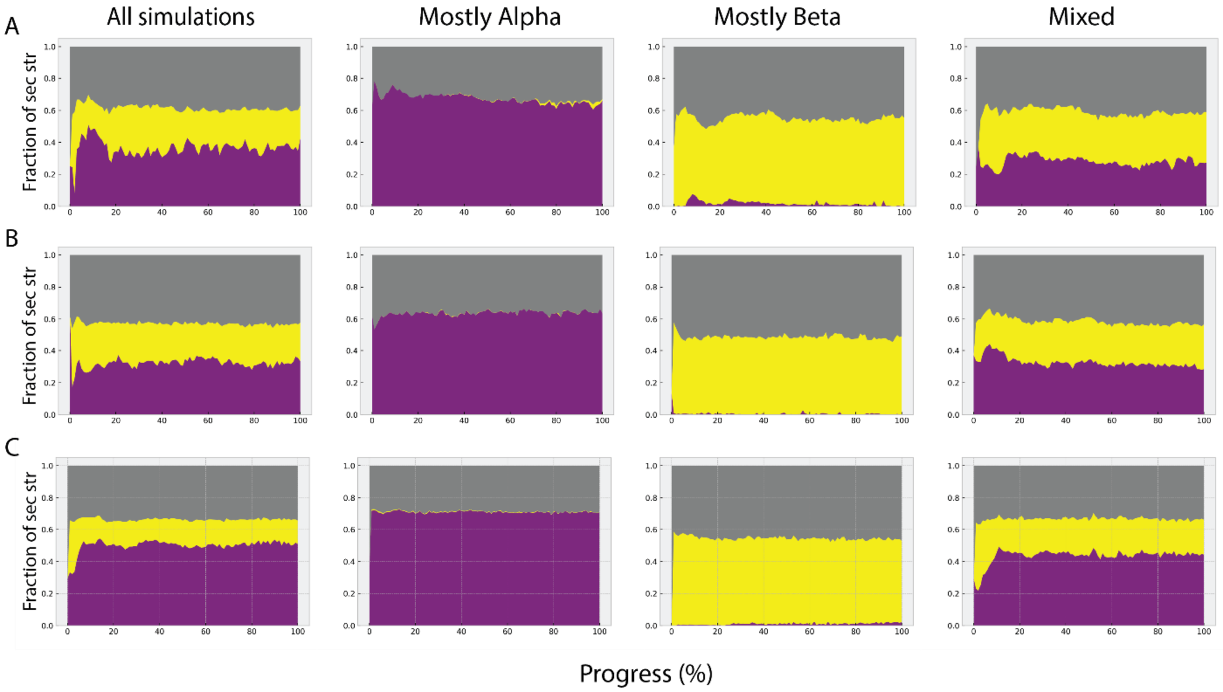

Figure S6. Changes in the secondary structure composition are averaged over all simulations and separately for mostly alpha, mostly beta, and mixed folds. The lengths of different lineages were normalized and are presented as progress. (A) Simulations with indels and a population size of 100. (B) Simulations with fixed length and population size of 100. (C) Simulations with fixed length and population size of 1000. See Table 1 for details.

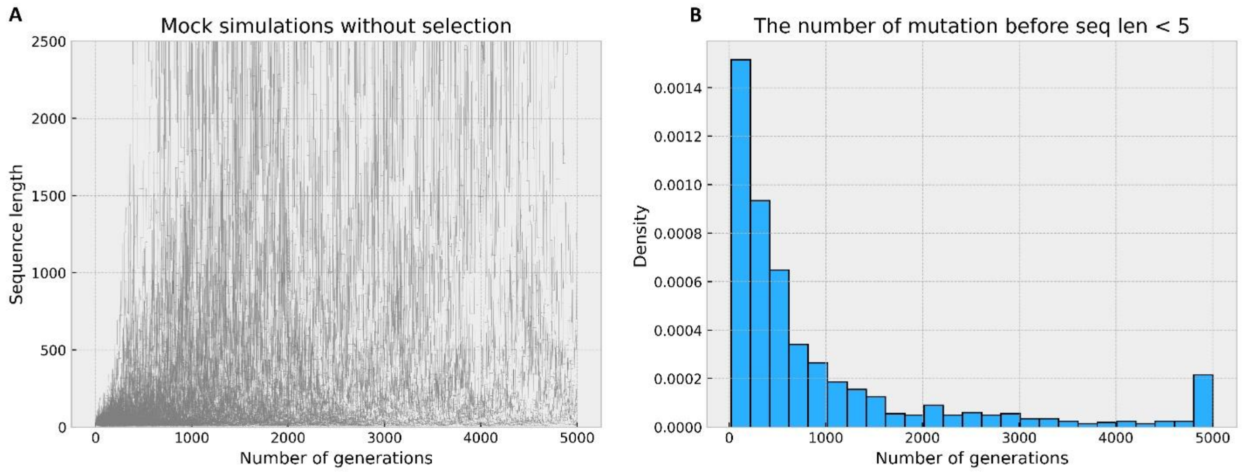

Figure S7. PFES without selection demonstrating changes in the protein length driven solely by the rates of indels presented in Table S2. **(A)** Simulations showing the length of sequences in the absence of any selective pressure, n=1000, starting sequence length = 24. **(B)** Distribution of the number of mutations before reaching the sequences sequence length of < 6 residues.

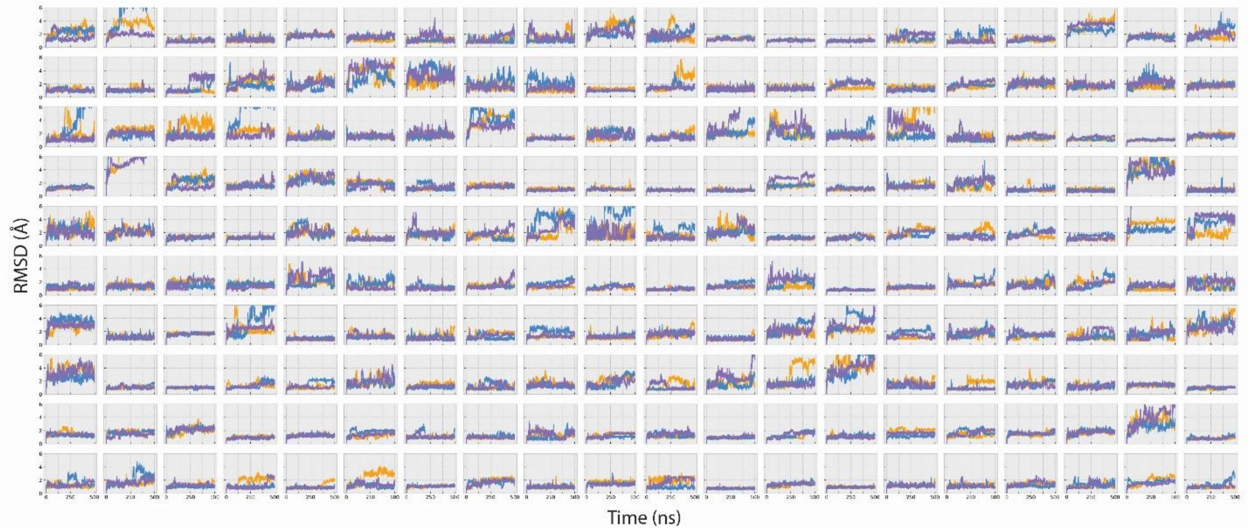

Figure S8. Backbone RMSD fluctuations for all proteins (n=200) measured after 3x MD simulations for each protein. Plots are ordered in the same way as proteins in Figure 2E.

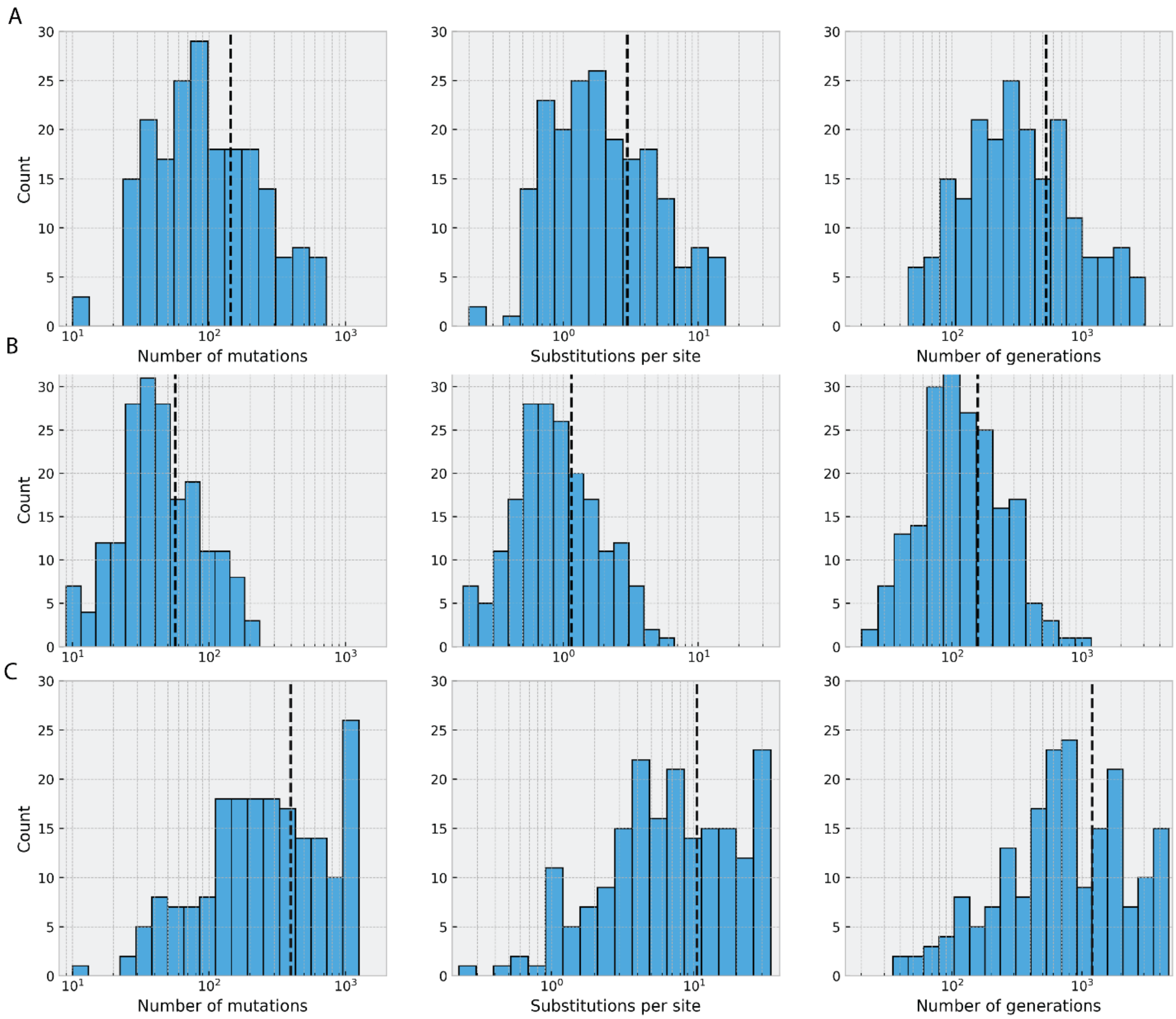

Figure S9. Number of mutations to fold nucleation estimated from simulations with fixed chain length or with indels. The data are presented as histograms on a log scale. The dashed line shows the mean of the distribution. (A) Simulations with indels and a population size of 100. (B) Simulations with a fixed length and population size of 100. (C) Simulations with fixed length and population size of 1000. See Table 1 for details. The curves in Figure 4 were obtained by kernel density estimation (KDE) of the same histograms on the natural scale.

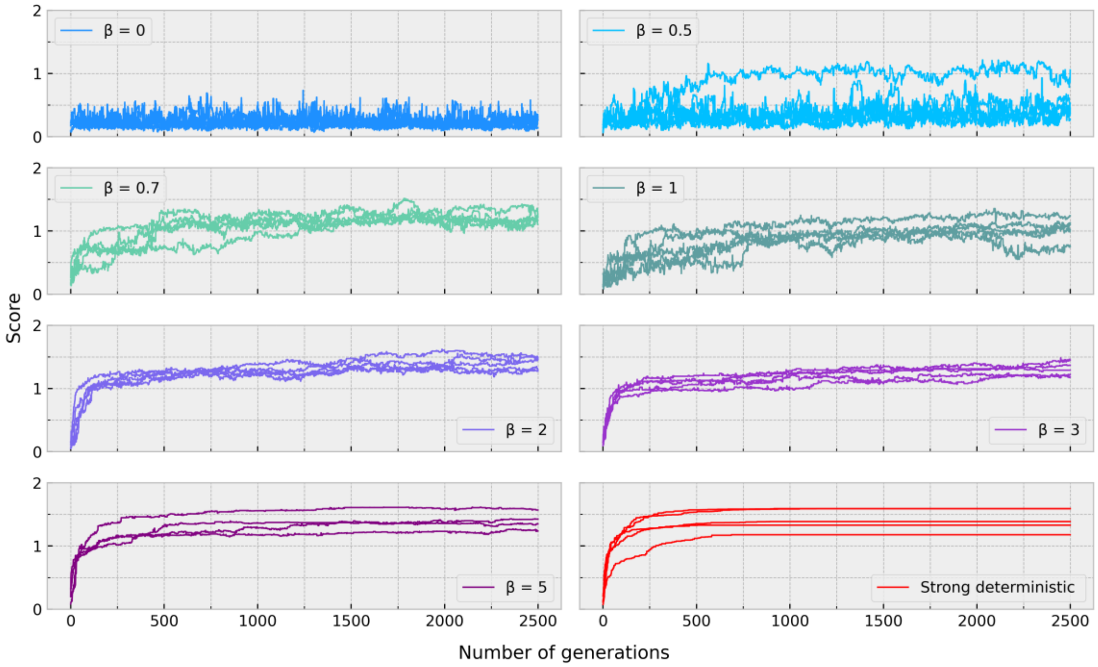

Figure S10. Simulation with stochastic selection of a 50 amino acid random peptide with different sampling temperatures (β).

Table S1. Foldseek search results

query	target	prob	fident	qcov	alnlen	evalue	db	nhits
run105	3vu4_B	1.00	0.08	0.90	73	0.00033	pdb	2640
run48	7vfk_B	1.00	0.12	0.96	92	0.00180	pdb	2023
run69	7vfm_C	1.00	0.15	0.92	73	0.00190	pdb	2010
run99	4x7r_A	1.00	0.19	0.82	52	0.00254	pdb	1912
run124	2q4m_A	1.00	0.11	0.98	143	0.00350	pdb	675
run18	4dl0_C	1.00	0.13	0.99	71	0.00748	pdb	1071
run93	7vfk_B	1.00	0.10	0.94	68	0.00834	pdb	1995
run188	5c0m_A	1.00	0.18	0.98	57	0.00996	pdb	2001
run22	5lhz_C	1.00	0.17	1.00	52	0.09474	pdb	818
run100	6htj_B	1.00	0.19	0.97	37	0.25540	pdb	1699
run130	6hhm_A	0.99	0.04	0.82	45	0.61880	pdb	464
run87	5twb_A	0.99	0.09	0.84	32	0.45460	pdb	653
run185	5twb_A	0.99	0.12	0.92	34	0.46980	pdb	776
run16	6y2s_A	0.99	0.13	0.83	45	0.53680	pdb	580
run77	7ywp_A	0.99	0.11	0.94	84	0.04018	pdb	417
run133	6czi_B	0.99	0.16	0.96	100	0.04278	pdb	111
run141	3bk5_A	0.98	0.10	0.96	48	0.94090	pdb	519
run50	3u8v_B	0.98	0.11	0.87	62	0.62310	pdb	132
run80	2mm3_A	0.98	0.13	0.94	46	0.67640	pdb	419
run31	5uw7_A	0.98	0.09	0.98	98	0.04906	pdb	304
run156	6ttu_T	0.98	0.14	0.97	77	0.30910	pdb	266

run5	2ecf_A	0.97	0.19	0.98	48	0.45540	pdb	437
run38	7l9e_E	0.96	0.14	0.95	59	0.49280	pdb	495
run194	AF-A0A4Q2RST3	1.00	0.22	1.00	69	0.00093	afdb	651
run41	AF-A0A143G9F3	1.00	0.21	0.86	61	0.00479	afdb	376
run193	AF-A0A814L140	1.00	0.15	1.00	72	0.02876	afdb	389
run96	AF-A0A2D3PMG7	1.00	0.08	0.95	89	0.03642	afdb	131
run199	AF-A0A7W0LT51	1.00	0.29	0.89	42	0.04865	afdb	816
run146	AF-A0A7S4L576	1.00	0.21	0.95	57	0.05781	afdb	39
run95	AF-A0A7C7HK81	1.00	0.20	0.88	50	0.07864	afdb	581
run165	AF-A0A2D8AN05	1.00	0.28	0.93	39	0.14870	afdb	174
run197	AF-A0A0F9JEQ6	1.00	0.16	1.00	56	0.17330	afdb	90
run90	AF-A0A7W7KAE5	1.00	0.21	0.88	43	0.17680	afdb	96
run176	AF-W7Z7K7	1.00	0.16	0.97	38	0.17950	afdb	499
run180	AF-A0A0C2W3X4	1.00	0.05	0.92	43	0.19380	afdb	609
run187	AF-A0A7Y2Y380	1.00	0.11	0.98	45	0.23340	afdb	951
run139	AF-A0A5E4I478	1.00	0.19	0.96	43	0.26220	afdb	1040
run106	AF-A0A1Q2CMH5	1.00	0.24	0.96	42	0.31340	afdb	1174
run170	AF-A0A3M1T0E8	1.00	0.12	1.00	66	0.31390	afdb	268
run102	AF-A0A495M102	1.00	0.20	0.95	35	0.35360	afdb	31
run92	AF-A0A7Z9JEL9	1.00	0.15	0.95	60	0.55210	afdb	77
run178	AF-A0A3D8T9U8	1.00	0.21	0.81	43	0.61670	afdb	74
run111	AF-A0A3B6THA5	1.00	0.16	0.96	45	0.50580	afdb	21
run171	AF-A0A7X7Q3P4	1.00	0.18	0.85	33	0.52200	afdb	125
run3	AF-A0A7X1E4C3	1.00	0.18	0.98	87	0.02829	afdb	9
run115	AF-A0A5C7PGG2	1.00	0.12	0.89	33	1.02200	afdb	9
run107	AF-X0SW97	1.00	0.21	0.83	47	0.89180	afdb	59
run186	AF-A0A3S9TJV0	1.00	0.10	0.98	83	1.00600	afdb	7
run42	AF-A0A842B3M2	1.00	0.11	1.00	45	1.20700	afdb	3
run29	AF-A0A0E9NQ65	0.99	0.21	0.90	103	0.00134	afdb	1
run196	AF-A0A242WAS5	0.99	0.18	0.93	57	0.23890	afdb	16
run140	AF-A0A414NWH6	0.99	0.28	0.97	36	2.30300	afdb	23
run151	AF-A0A521K8B3	0.99	0.33	0.91	40	0.54510	afdb	47
run131	AF-A0A2R6RQF2	0.99	0.28	0.93	39	1.18800	afdb	6
run108	AF-A0A7C4TWM1	0.99	0.21	0.92	56	1.62600	afdb	2
run190	AF-A0A1Q4GU02	0.99	0.15	0.83	41	1.07700	afdb	36
run198	AF-F4P5B8	0.99	0.19	0.96	53	0.66070	afdb	21
run36	AF-A0A3P6B3K0	0.99	0.21	0.88	43	2.00800	afdb	15
run53	AF-T1XNQ2	0.99	0.12	0.96	82	2.01300	afdb	3
run25	AF-A0A849ZRA1	0.98	0.14	0.90	43	3.90900	afdb	1
run129	AF-I1IM41	0.98	0.19	0.91	42	4.24100	afdb	1
run157	AF-A0A517TSJ5	0.98	0.17	0.91	78	1.59100	afdb	2
run66	AF-S1N7D9	0.98	0.17	0.96	53	4.10100	afdb	10
run83	AF-A0A537X4E9	0.98	0.14	1.00	88	0.28350	afdb	5
run47	AF-A0A316YHF2	0.98	0.14	0.96	90	0.37140	afdb	7
run128	AF-A0A1Q6UZD7	0.98	0.17	0.84	36	1.77100	afdb	13
run159	AF-A0A535GJU4	0.97	0.08	0.93	52	3.79500	afdb	5

run168	AF-M1VDQ2	0.97	0.21	0.88	53	4.11700	afdb	3
run110	AF-A0A239QJR5	0.97	0.21	0.83	34	4.81300	afdb	1
run64	AF-A0A429CVJ2	0.96	0.25	0.95	56	2.36200	afdb	1
run26	AF-A0A7K4DWC2	0.96	0.07	0.89	68	3.39600	afdb	1
run86	AF-A0A7J0EWZ7	0.96	0.17	0.98	41	0.86610	afdb	4
run109	AF-A0A418PM05	0.96	0.25	0.82	28	4.14700	afdb	2
run32	MGYP000472187349	0.99	0.15	0.92	62	0.17890	esm	1
run72	MGYP001476248561	0.99	0.16	0.92	55	1.59300	esm	4
run88	MGYP003324074694	0.98	0.20	0.95	41	0.24300	esm	2
run94	MGYP001385367156	0.98	0.14	0.95	78	0.70690	esm	1
run52	MGYP003295344537	0.98	0.18	0.91	40	1.33500	esm	1
run104	MGYP003590916796	0.98	0.16	0.98	62	0.47420	esm	1
run154	MGYP003706959411	0.96	0.16	0.97	69	1.54600	esm	1
run79	MGYP003353214145	0.96	0.13	0.87	32	1.75000	esm	1

rep target – a representative target for this query; prob – foldseek probability; fident – fraction of identical matches; qcov – query coverage, alnlen – alignment length; db – database where the representative hit is coming from; nhits – total number of hits

Table S2. Compleat list of Foldseek hits.

Table S3. Mutation rates used in PFES.

Mutation	Symbol	Flat rate	Codon rates	Uniprot rate
Alanine	A	1	1.311	1.652
Cyste`ine	C	1	0.656	0.278
Aspartate	D	1	0.656	1.092
Glutamate	E	1	0.656	1.344
Phenylalanine	F	1	0.656	0.774
Glycine	G	1	1.311	1.414
Histidine	H	1	0.656	0.456
Isoleucine	I	1	0.984	1.182
Lysin	K	1	0.656	1.16
Leucine	L	1	1.967	1.93
Methionine	M	1	0.328	0.482
Asparagine	N	1	0.656	0.812
Proline	P	1	1.311	0.95
Glutamine	Q	1	0.656	0.786
Arginine	R	1	1.967	1.106
Serine	S	1	1.967	1.33
Threonine	T	1	1.311	1.072
Valin	V	1	1.311	1.372
Tryptophan	W	1	0.328	0.22
Tyrosine	Y	1	0.656	0.584
Single residue insertion	+	1	1	1
Single residue deletion	-	1	1	1
Partial duplication	*	0.4	0.4	0.4
Random insertion	/	0.4	0.4	0.4
Partial deletion	%	0.9	0.9	0.9
Circular permutation	p	0.1	0.1	0.1
Full duplication	d	0.05	0.05	0.05
